# Immune Recognition of Citrullinated Proteoglycan Aggrecan Epitopes in Mice with Proteoglycan-Induced Arthritis and in Patients with Rheumatoid Arthritis

**DOI:** 10.1371/journal.pone.0160284

**Published:** 2016-07-28

**Authors:** Adrienn Markovics, Tímea Ocskó, Robert S. Katz, Edit I. Buzás, Tibor T. Glant, Katalin Mikecz

**Affiliations:** 1 Section of Molecular Medicine, Department of Orthopedic Surgery, Rush University Medical Center, Chicago, Illinois, United States of America; 2 Rheumatology Associates, Rush University Medical Center, Chicago, Illinois, United States of America; 3 Department of Genetics, Cell and Immunobiology, Semmelweis University, Budapest, Hungary; Faculté de médecine de Nantes, FRANCE

## Abstract

**Background:**

Rheumatoid arthritis (RA) is an autoimmune inflammatory disease affecting the joints. Anti-citrullinated protein antibodies (ACPA) are frequently found in RA. Previous studies identified a citrullinated epitope in cartilage proteoglycan (PG) aggrecan that elicited pro-inflammatory cytokine production by RA T cells. We recently reported the presence of ACPA-reactive (citrullinated) PG in RA cartilage. Herein, we sought to identify additional citrullinated epitopes in human PG that are recognized by T cells or antibodies from RA patients.

**Methods:**

We used mice with PG-induced arthritis (PGIA) as a screening tool to select citrulline (Cit)-containing PG peptides that were more immunogenic than the arginine (R)-containing counterparts. The selected peptide pairs were tested for induction of pro-inflammatory T-cell cytokine production in RA and healthy control peripheral blood mononuclear cell (PBMC) cultures using ELISA and flow cytometry. Anti-Cit and anti-R peptide antibodies were detected by ELISA.

**Results:**

Splenocytes from mice with PGIA exhibited greater T-cell cytokine secretion in response to the Cit than the R version of PG peptide 49 (P49) and anti-P49 antibodies were found in PGIA serum. PBMC from ACPA+ and ACPA- RA patients, but not from healthy controls, responded to Cit49 with robust cytokine production. High levels of anti-Cit49 antibodies were found in the plasma of a subset of ACPA+ RA patients. Another PG peptide (Cit13) similar to the previously described T-cell epitope induced greater cytokine responses than R13 by control (but not RA) PBMC, however, anti-Cit13 antibodies were rarely detected in human plasma.

**Conclusions:**

We identified a novel citrullinated PG epitope (Cit49) that is highly immunogenic in mice with PGIA and in RA patients. We also describe T-cell and antibody reactivity with Cit49 in ACPA+ RA. As citrullinated PG might be present in RA articular cartilage, Cit PG epitope-induced T-cell activation or antibody deposition may occur in the joints of RA patients.

## Introduction

Rheumatoid arthritis (RA) is a chronic autoimmune disease characterized by inflammatory destruction of the peripheral joints. It affects approximately 1% of the adult human population with a female preponderance. The etiology of RA is unknown, although several lines of evidence suggest that genetic and environmental factors play an important role in the development of the disease [1]. A strong genetic linkage exists between disease susceptibility and MHC class II (HLA-DRB1) alleles expressed by antigen-presenting cells, indicating the involvement of T cells in RA pathogenesis [1][2]. Most patients with RA produce autoantibodies (autoAbs) against self-IgG (rheumatoid factor) and/or several native or citrullinated self-proteins (anti-citrullinated protein Abs, ACPA) [3]. Citrullination is a post-translational protein modification catalyzed by peptidyl arginine deiminase (PAD) enzymes, which convert protein-bound arginine to citrulline. Citrullination of various proteins occurs under both physiologic and pathologic conditions (reviewed in [4]). Although the arginine to citrulline conversion leads to creation of “neoepitopes”, production of autoAbs against citrullinated neoepitopes (i.e., ACPA) is highly specific for RA [5][6]. Based on the presence of ACPA in the serum, the onset of RA [7][8] and disease progression [9][10] can be predicted. Moreover, the presence of ACPA in the serum of patients, which is usually detected by anti-cyclic citrullinated peptide (anti-CCP) Ab assays, is listed among the disease-specific serological markers in the 2010 classification criteria of RA [11]. The wide repertoire of ACPA-reactive citrullinated self-proteins includes those found in joint tissues such as cartilage-specific type II collagen (CII), fibrinogen, vimentin, and histones, but citrullinated epitopes in some joint-unrelated proteins can be recognized by ACPA [5][6]. Immune complexes formed between citrullinated autoantigens (e.g., citrullinated vimentin, fibrinogen, and others) and ACPA have been identified in RA patients and are thought to play an important role in the pathogenesis of RA by triggering or perpetuating inflammatory processes within the joint [12–15].

The large aggregating proteoglycan (PG) of cartilage (also termed aggrecan) is one of the major macromolecules of the extracellular matrix of articular cartilage. Similarly to CII, which can elicit collagen-induced arthritis (CIA) in DBA/1 mice [16], immunization with PG can induce chronic inflammatory arthritis (PGIA) in genetically susceptible BALB/c mice [17][18]. Our previous epitope-mapping studies identified immunodominant T-cell epitopes within the first globular (G1) domain of human PG [19][20], among which the so-called 5/4E8 epitope (ATEGRVRVNSAYQDK) was clearly associated with T-cell activation and arthritogenicity in BALB/c mice. A construct of a T-cell receptor (TCR) recognizing this epitope was used to create PG-specific TCR transgenic (PG-TCR tg) mice, which developed aggravated arthritis upon immunization with human PG [21]. The arthritogenic potential of the 5/4E8 and other epitopes of the PG G1 domain was further confirmed by the observation that immunization of BALB/c mice with the recombinant G1 domain of human PG (rhG1) induced arthritis (termed G1-induced arthritis or GIA) with clinical and immunological features similar to, but not identical with, those in PGIA [22]. Intriguingly, anti-CCP Abs (ACPA) could be detected in the sera of mice with PGIA, and, at much lower levels, in the sera of mice with GIA [22]. Moreover, we recently reported that cartilage extracts from patients with RA or osteoarthritis (OA) contained PG molecules reactive with ACPA+ RA serum [23]. Removal of the PG G1 domain from the cartilage extracts by immuno-absorption dramatically reduced ACPA reactivity, suggesting that the G1 domain of PG contained most of the citrulline residues recognized by ACPA [23].

An extended and citrullinated (Cit) version of the 5/4E8 peptide (VVLLVATEGCitVRVNSAYQDK) was tested in two independent laboratories for induction of T-cell responses in RA patients. The first study [24] found that this citrullinated PG peptide induced robust in vitro production of interleukin (IL)-17 by T cells from the majority of RA patients tested, but not by T cells from healthy controls. The second study [25] reported increased in vitro production of IL-6 in the presence of this citrullinated PG peptide by cells from RA patients and even by cells from some of the healthy controls. The native (arginine-containing) version of the same peptide did not induce T-cell responses in either study [24][25]. Interestingly, we found that only the native form of the 5/4E8 peptide, but not its citrullinated versions, induced proliferation and pro-inflammatory cytokine responses in T cells from mice with PGIA [26]. Another citrullinated peptide from the G1 domain of PG (AGWLACitRSVRYPI), but not its arginine-containing counterpart, was found by Aggarwal et al [27] to induce in vitro proliferation of peripheral blood mononuclear cells (PBMC) of RA patients. Unfortunately, sequence alteration of the peptides used is a confounding factor in the interpretation of the results of that study [27].

In summary, T-cell reponses against the citrullinated form of an immunodominant peptide from the PG G1 domain have been reported in RA patients. However, the G1 domain contains 23 arginine residues, any of which may be converted to citrulline, and it is not known if RA T cells can recognize additional citrullinated epitopes within the G1 domain of human PG. It is also unknown whether T-cell recognition of any citrullinated G1 peptide is accompanied by the presence of ACPA against the same or other peptide in the circulation of a given patient. Therefore, the major goal of this study was to map the entire G1 domain of human PG for citrullinated epitopes preferentially recognized (relative to the corresponding native epitope) by T cells or Abs from RA patients. Our peptide library included 64 pairs of native (arginine-containing: R) and citrullinated (citrulline-containing: Cit) peptides. Mapping of individual epitopes represented by these 128 peptides would have required very high numbers of human PBMC. In order to reduce the number of primary human cells necessary for these studies, we took two steps: First, we sorted the PG peptides into 16 R and 16 Cit peptide pools, and used mice with PGIA as screening tools for the selection of a limited number of individual Cit peptides preferentially recognized by T cells from those mice. Second, we expanded the T cells in human PBMC cultures between two rounds of peptide stimulation. To determine if preferential T-cell responses to any Cit peptide were accompanied by preferential Ab reactivity with the same or other Cit PG peptides in mice or humans, we performed anti-PG peptide Ab assays using mouse serum and human plasma samples. As there is ample evidence of PG-specific immune responses in mice with PGIA or GIA [18][20][22] and some evidence of Cit PG-specific immunity in RA patients [23–25], we also sought to uncover similarities in the immune recognition pattern of PG epitopes between mice with PGIA and ACPA+ RA patients, between mice with GIA and ACPA- RA patients, as well as between naïve mice and healthy control human subjects.

## Materials and Methods

### Construction of a human PG peptide library

**Fig 1:**
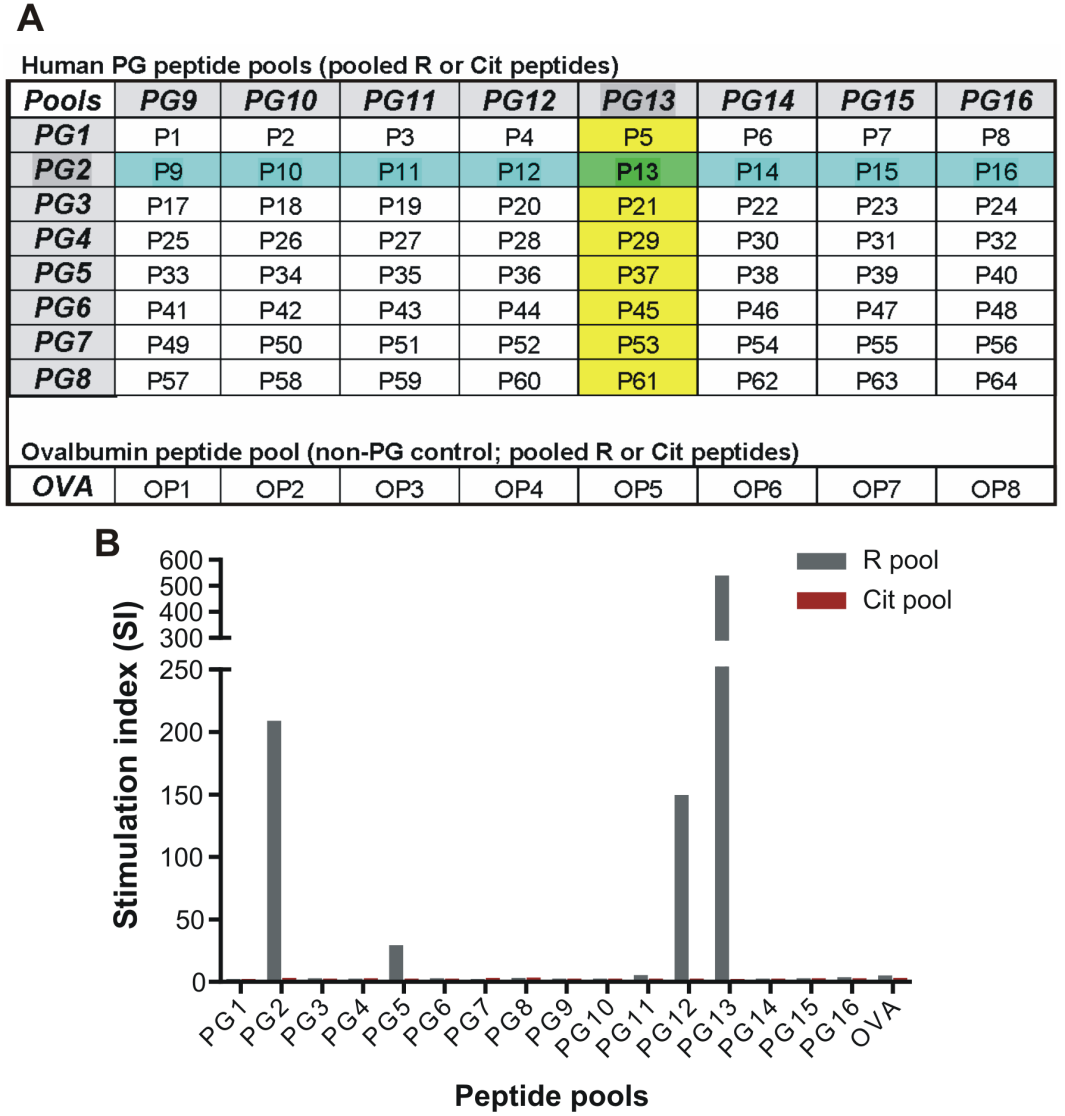
Illustration and validation of the peptide pooling strategy. (A) Arginine (R) and citrulline (Cit)-containing peptide pairs representing human proteoglycan (PG) sequences (S1 Table) were sorted into 16 pairs of PG peptide pools (PG1-16), with each pair containing 8 individual R or Cit peptides. Each individual R or Cit peptide was represented in 2 different R or Cit pools. For example, R-Cit peptide pair 13 (P13, highlighted in green) is represented in both the PG2 R-Cit pools (highlighted in blue) and the PG13 R-Cit pools (highlighted in yellow). Eight R- and 8 corresponding Cit peptide pairs (OP1-8) representing chicken egg ovalbumin were used as non-PG peptide controls and were pooled in an R and a corresponding Cit pool (OVA). (B) The PG peptide pooling strategy was validated using spleen cells from PG-specific T-cell receptor transgenic (PG-TCR tg) mice. T cells of PG-TCR tg mice recognize the R version of the P13 peptide. As shown by the stimulation index (SI) of cell proliferation, PG-TCR tg cells (mixed from 2 spleens) proliferated vigorously in the presence of R pools of PG2 and PG13 and to a lesser extent, in the presence of the R pool of PG12 containing the R version of peptide P12 (having 80% overlap with the R version of peptide P13). The cells did not recognize the Cit pools of PG2 and PG13 or any other peptide pool not containing the R version of P13 or P12. SI was calculated as described in the Methods. Data shown are the mean SI of cells in triplicate wells.

Peptide sequences were selected from the arginine-containing regions of the G1 domain and a short segment of the neighboring interglobular domain of human cartilage PG aggrecan. The overlapping sequences were 15 amino acid (aa)-long with 12-mer overlap. Native (arginine-containing) and corresponding citrullinated (citrulline-substituted) peptides were synthesized by Biomatik (Wilmington, DE, USA). The libraries of 64 arginine-containing (R) and 64 citrulline-containing (Cit) peptides (sequences are shown in S1 Table) were sorted into 16 R and 16 Cit pools of 8 peptides so that each peptide was represented in 2 different pools (R-Cit pairs of peptide pools are shown as PG1-16, and individual R-Cit peptide pairs are indicated as P1-64 in Fig 1A). The non-PG control peptide pool contained 8 randomly chosen R (and corresponding Cit) peptides from chicken egg ovalbumin (OVA). Following selection on the basis of T-cell reactivity with R and Cit PG peptides in mice, 6 pairs of individual R and Cit PG peptides were synthesized with a biotin tag at the N terminus for anti-PG peptide Ab ELISA assays.

### Mice, immunization, and assessment of arthritis

Adult female BALB/c mice were purchased from the Frederick colony of Charles River (Wilmington, MA, USA). Transgenic BALB/c mice, expressing a PG-specific TCR (PG-TCR tg) have been characterized previously [21]. PG was extracted from human articular cartilage as described in detail elsewhere [17][28–30]. Collection of cartilage from OA patients who provided signed written consent before undergoing knee joint replacement surgery was organized through the Orthopedic Information, Tissue and Implant Repository Study, approved by the Institutional Review Board (IRB) of Rush University Medical Center, Chicago, IL, USA (IRB approval number L00011021). To induce PGIA, wild-type BALB/c mice were immunized intraperitoneally with human OA PG (100 μg protein) [29–31] emulsified in dimethyl-dioctadecyl ammonium bromide adjuvant (DDA; Sigma-Aldrich, St Louis, MO, USA) three times, 3 weeks apart [30][32]. The limbs of mice were inspected for signs of arthritis (swelling and redness) 3 times a week after the second PG immunization. The degree of arthritis was scored visually on a scale of 0 to 4 for each limb (score 0: no swelling or redness; score 1: mild swelling of the distal paw or at least 2 digits; score 2: moderate swelling of the paw joints distal to the ankle or wrist; score 3: pronounced swelling and redness of the entire paw including the ankle or wrist; score 4: severe swelling and redness of the entire paw with joint stiffness) [30–32]. Recombinant human PG G1 domain (rhG1) protein was produced as described previously [22]. Groups of wild-type BALB/c mice were immunized three times with 40 μg rhG1 in DDA, and the limbs of animals with GIA [22] were scored for the degree of inflammation as described for PGIA. At the onset of arthritis, rodent food was placed at the bottom of the cages to ensure that the animals could access food without climbing. Mice were sacrificed as soon as they reached the acute phase of arthritis (14–15 days after the 3^rd^ immunization). Before sacrifice, mice were anesthetized by intramuscular injection of a mixture of ketamine (100 mg/kg) and xylazine (8 mg/kg) and were bled for collection of serum samples. Euthanasia was carried out through carbon dioxide inhalation. All experiments involving animals were reviewed and approved by the Institutional Animal Care and Use Committee (IACUC) of Rush University Medical Center (IACUC permit number 14–001).

### Mouse cell cultures

Spleens were harvested from BALB/c mice with PGIA or GIA, or from naïve BALB/c mice. Spleen cells from naïve PG-TCR tg mice whose T cells recognize the sequence ATEGRVRVNSAYQDK (peptide R13 in S1 Table) [21] were used as reference/peptide specificity controls. Cells were released by pressing the spleens under sterile metal screens and erythrocytes were eliminated by hypotonic lysis. The spleen cells were suspended in Dulbecco’s Modified Eagle Medium (DMEM; Sigma-Aldrich) containing antibiotics/antimycotics and supplemented with 10% fetal bovine serum (FBS; Hyclone, Logan, UT, USA). The cells were dispensed into 96-well culture microplates (2.5 x 10^5^ viable cells in 200 μl medium per well). R and Cit peptides were dissolved in dimethyl sulfoxide or sterile acetonitrile-in-water (40%/60% vol/vol) solvent at a concentration of 10 μg/μl of each of the peptides, and 8 μl of a pool of 8 peptides was added to the cells in triplicate wells. Control wells contained no peptide (only solvent) or the OVA peptide pool. Occasionally, sterile human PG (25 μg/well) or rhG1 (7.5 μg/well) was also included as a positive reference control. The cells were cultured in the absence or presence of peptide pools or control materials for 5 days for determination of cell proliferation. The cells were pulsed with 0.5 μCi/well of [^3^H] thymidine (Perkin Elmer, Boston, MA, USA) 18–20 hours before harvest. Isotope incorporation was measured using a scintillation counter (Micro-Beta; Perkin Elmer) and expressed as counts per minute (cpm). Stimulation index (SI) was calculated by dividing the cpm values of peptide-containing or positive control wells with the cpm of wells containing no peptide. Cit:R ratio, i.e., stimulation by Cit PG peptide pools relative to R PG peptide pools was calculated by dividing the SI value of each Cit peptide pool with the SI value of the corresponding R peptide pool. Individual peptide pairs (P) for further testing were selected on the basis of their presence in 2 PG peptide pools that yielded a Cit:R ratio of greater than 1.0.

Spleen cell cultures from PGIA mice were also set up separately for determination of peptide-induced cytokine production. For these experiments, spleen cells were dispensed into 48-well plates (3x10^6^ cells in 900 μl volume per well) and cultured in the absence or presence of pre-selected individual R and Cit peptides (40 μg/ml each). On day 4 of culture, the supernatants were collected for measurement of soluble cytokine concentrations.

### Cytokine ELISA

Concentrations of IL-17, interferon (IFN)γ, IL-6, and IL-10 in the mouse spleen cell culture supernatants were measured using murine cytokine ELISA kits (R & D Systems, Minneapolis, MN, USA), according to the manufacturer’s instructions. Briefly, 96-well Maxisorp ELISA plates (Thermo Scientific, Rochester, NY, USA) were coated with the purified anti-cytokine capture Ab at the recommended concentration overnight. After repeated washing, free binding sites were blocked with a blocking buffer containing 1% bovine serum albumin (BSA; Sigma-Aldrich). Undiluted cell culture supernatants (50–100 μl/well) and serially diluted cytokine standards were then incubated in duplicate wells with the immobilized capture Ab for 2 hours. After repeated washing, biotinylated anti-cytokine detection antibody was applied to the wells followed by horseradish peroxidase (HRP)-conjugated streptavidin. The color reaction was developed with 3,3',5,5'-tetramethylbenzidine substrate (BD Biosciences, San Diego, CA, USA). Optical density (OD) at 450 nm, and corresponding cytokine concentrations were determined using a Synergy 2 ELISA reader (BioTek Instruments, Winooski, VT, USA).

### Patients

Forty six patients diagnosed with RA according to the 2010 American College of Rheumatology/European League Against Rheumatism classification criteria [11] and 9 healthy control subjects participated in the study by donating blood. Signed written informed consent was obtained from all participants at Rheumatology Associates, Rush University Medical Center, and the study was approved by the IRB of Rush University Medical Center (IRB approval number L89050101). Of the 46 patients with RA, 34 were ACPA+ and 12 were ACPA- as determined by anti-CCP assay (CCP3 kit, Inova Diagnostics, San Diego, CA, USA). The average age of patients was 55.2 years, 30 were females and 16 were males. The average age of healthy controls (HC) was ~52 years, 6 were females and 3 were males. None of the participating RA patients received B cell-depleting therapy (e.g., Rituximab).

### Human cell cultures

Peripheral blood from each human subject was collected in heparinized tubes, and PBMC were separated on a Ficoll-Paque PLUS density gradient (GE Healthcare Life Sciences, Marlborough, MA, USA) according to a standard protocol pertaining to Ficoll separation. Before harvesting the PBMC layer, 3–4 ml plasma was collected from each sample and stored at -20°C until use. The cells were washed with DMEM, suspended in DMEM supplemented with 10% FBS, and dispensed into 48-well plates at 1x10^6^ cells in 300 μl or 2−10^6^ cells in 600 μl per well, depending on the yield of viable cells from each subject. The cells were cultured in the presence of individual R and Cit peptides (40 μg/ml) for 5 days. Control wells contained solvent only without peptide. Surviving (mainly peptide-responsive) T cells in the cultures were then expanded by addition of 40 ng/ml recombinant human/murine IL-2 (PeproTech, Rocky Hill, NJ, USA) for another 5–6 days with medium change and addition of fresh IL-2 at least once during the expansion period. IL-2 was then removed by extensive washing, and the cells were re-stimulated with the original R and Cit peptides for 3 days. Cells in wells not containing peptide in the first round of culture were treated with solvent only in the final round. At the end of this 13-14-day culture period, supernatants were collected for soluble cytokine assays and the cells were harvested for flow cytometry (see below).

Concentrations of IL-17, IFNγ, and IL-6 in the human cell culture supernatants were measured using human cytokine ELISA kits (R & D Systems). The assays were carried out according to the manufacturer’s specific instructions for each kit. The general assay procedures and measurements of cytokine concentrations in the human cell culture supernatants were the same as described for the murine cytokine ELISA above.

### Flow cytometry

Flow cytometric detection of intracellular IL-17 and IFNγ in human PBMC was carried out after an extended period of culture as described above. After 13–14 days of culture in the absence or presence of R and corresponding Cit peptides, a staining protocol and a fixation/permeabilization kit (Cytofix/Cytoperm kit with GolgiStop) from BD Biosciences were employed to detect intracellular cytokines as described previously [32]. In brief, the cells (2 x 10^6^/ml culture medium) were first incubated with 10 ng/ml phorbol-13-myristate acetate (PMA, Sigma-Aldrich), 1 μg/ml ionomycin (Invitrogen, Grand Island, NY, USA), and 1 μl/ml GolgiStop (containing 2 μM monensin) for 4 hours. After blocking Fc receptors with a mouse anti-human CD16/32 mAb, followed by surface staining with a fluorochrome-conjugated mouse mAb against human CD4 (BioLegend, San Diego, CA, USA), the cells were fixed, permeabilized, and stained with fluorochrome-conjugated mouse mAbs to human IL-17A and IFNγ (BioLegend). Flow cytometry was performed using a BD FACS Canto II instrument, and data were analyzed with FACS Diva software (BD Flow Cytometry Systems, San Jose, CA, USA). Non-specific background fluorescence was determined by staining a separate aliquot of human cells with mouse IgG isotype controls (BioLegend) matching the isotypes and fluorochromes of the CD4- and cytokine-specific mAbs.

### Measurement of anti-PG peptide Abs in mouse serum and human plasma

Anti-peptide IgG Abs in mouse serum samples were detected using an in-house ELISA system employing neutravidin-coated 96-well plates pre-blocked with BSA (Pierce, Rockford, IL, USA) and biotinylated R or Cit peptides for Ab capture. In brief, 10 mg/ml stock solutions of the biotinylated peptides were diluted to 25 μg/ml in Tris-buffered saline (25 mM Tris, 150 mM NaCl; pH 7.2) containing 0.1% BSA and 0.05% Tween-20. Duplicate wells of neutravidin-coated and BSA pre-blocked plates were incubated with the biotinylated peptide solutions (100 μl/well) for 1 hour at room temperature. After repeated washing, mouse serum samples (at 1:1000 dilution) were added to the wells and incubated for 1 hour. Extensive washing was followed by detection of plate-bound mouse IgG with HRP-conjugated goat anti-mouse IgG Ab (BD Biosciences). The color reaction was developed and the OD values were read as described for the mouse cytokine ELISA. Background controls included wells treated with the biotinylated peptides and secondary anti-mouse IgG Ab without addition of mouse serum.

Anti-peptide Abs in human plasma (at 1:100 dilution) were measured in a similar manner as described for mouse serum, but peptide-bound IgG was detected with HRP-conjugated anti-human IgG (BD Biosciences). For both the mouse and human systems, delta (Δ)OD values were calculated after subtracting the background OD from the sample OD values. The ratio of Cit- and R-peptide-specific Abs was calculated by dividing the ΔOD of the Cit peptide-containing well with the ΔOD of the corresponding R peptide-containing wells.

### Statistical analysis

Data are presented as the mean or the mean±SEM. Data were analyzed using GraphPad Prism 6 statistical software (GraphPad, La Jolla, CA, USA). Ratios of cell or Ab reactivity with paired Cit and R peptides or peptide pools (Cit:R ratio relative to a ratio of 1.0) were analyzed using the Wilcoxon signed rank test. Data from two groups were compared using Mann-Whitney U test. Data from multiple groups were analyzed using Kruskal-Wallis test followed by Dunn’s test for multiple comparisons. Results affected by 2 factors were analyzed using two-way ANOVA followed by Tukey’s and Shidak’s tests for multiple comparisons. Correlation analysis was carried out using Pearson’s correlation test, and best-fit curves were created by linear regression. The actual tests used for statistical analysis and sample numbers are specified in the figure legends. Statistical significance was set at p<0.05 (two-tailed). For simplicity, all levels of statistical significance are indicated as p<0.05.

## Results and Discussion

### Validation of the peptide pooling strategy

Stimulation of spleen cells obtained from naïve PG-TCR tg mice with the 16 R PG peptide pools and 16 Cit PG peptide pools (and with non-PG OVA peptide pools) (Fig 1A) resulted in vigorous cell proliferation in wells containing the R versions of PG2, PG12, and PG13 peptide pools, as indicated by the high stimulation indices (Fig 1B). As the P13 peptide (highlighted in green in Fig 1A), which is specifically recognized by T cells from PG-TCR tg mice [21], was present in both the PG2 (highlighted in blue in Fig 1A) and PG13 (highlighted in yellow in Fig 1A) peptide pools, the result from PG-TCR tg mice confirmed the correct sequence of the R version of the P13 peptide (R13, S1 Table) and also validated the peptide pooling strategy. As expected, T cells from the PG-TCR tg mice did not proliferate in wells containing the Cit version of the P13 peptide (Cit13, S1 Table), as substitution of the first R with Cit likely destroyed the epitope of this TCR. PG-TCR tg cells also proliferated in the presence of the PG12 pool (Fig 1B), which could be explained by the 12 aa (80%) overlap between the R12 peptide (present in the PG2 and PG12 pools, Fig 1A) and the R13 peptide (S1 Table). Minimal or no proliferation of spleen cells from the naïve PG-TCR tg mice was detected in wells containing the rest of the PG R peptide pools or in those containing any of the PG Cit peptide pools or the OVA pools (Fig 1B).

### Determination of preferential proliferative responses to Cit PG peptide pools by mouse T cells and selection of “preferred” individual Cit peptides

**Fig 2:**
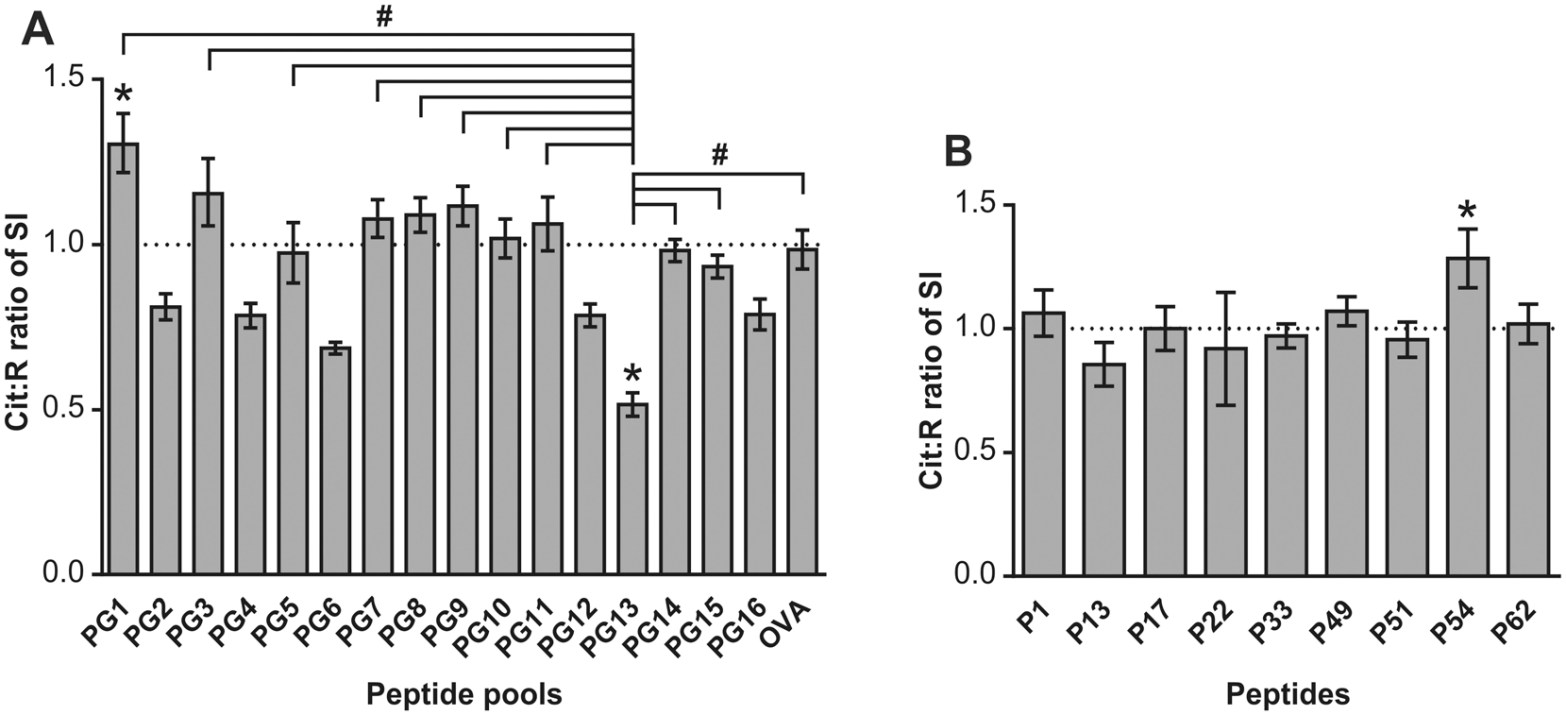
Proliferation of spleen cells from mice with PG-induced arthritis (PGIA) in response to stimulation with peptide pools and individual peptides. Spleen cells from mice with PGIA were cultured with (A) Cit and R pairs of PG or OVA peptide pools or (B) Cit and R pairs of selected individual peptides (P) in triplicate wells. The Cit:R ratio of SI was calculated as described in the Methods. Data are expressed as the mean of Cit:R ratio of SI±SEM (n = 12 mice). Equal response to the Cit and R version of a peptide pool or peptide (theoretical Cit:R ratio of 1.0, at which no preference for the Cit or R version is assumed) is indicated with a dotted line. Statistical analysis was performed using Wilcoxon signed rank test (*p<0.05: Cit:R ratio vs 1.0) and Kruskal-Wallis test followed by Dunn’s multiple comparison test (#p<0.05: Cit:R ratio of PG13 vs Cit:R ratio of any other peptide pool).

Using spleen cells from mice with PGIA, we tested the capacity of Cit and R PG peptide pools (16 each) as well as Cit and R OVA pools to induce in vitro proliferation. To determine which Cit PG pools were preferentially recognized, we calculated the Cit:R ratios of stimulation index values for each Cit-R pair of peptide pools. A Cit:R ratio of greater than 1.0 was considered as an indicator of preferential recognition of a PG Cit peptide pool over the corresponding PG R pool. As shown in Fig 2A, 7 of the 16 pairs of peptide pools (PG1, PG3, PG7, PG8, PG9, PG10, and PG11) yielded Cit:R ratios greater than 1.0, although the differences were modest and statistically significant only in the case of PG1 (*p<0.05). The remaining 9 pairs of PG peptide pools and the OVA pools produced Cit:R ratios below 1.0, with the PG13 pair having the lowest Cit:R ratio (*p<0.05) (Fig 2A). Poor recognition of the Cit version of the PG13 peptide pool relative to the R PG13 pool was not surprising, as the R pool of PG13 contained the R13 peptide that had been characterized as an immunodominant/arthritogenic T-cell epitope [19], while the Cit13 counterpart was not immunogenic in mice with PGIA [26]. Comparing the Cit:R ratio of the PG13 Cit and R pools with all the other pools, we found statistically significant differences in 10 pairs of PG pools and the OVA pool in favor of Cit pool recognition (#p<0.05)(Fig 2A). Proliferation assays performed in a similar manner, but using spleen cells from mice with GIA, revealed Cit:R ratios similar to those obtained in PGIA, although GIA cells exhibited preference for fewer Cit pools than cells from the PGIA group (S1 Fig). Overall, the proliferative responses to the first 13 PG peptide pools were similar in PGIA and GIA, further supporting the immunological similarities between these closely related models.

On the basis of cell proliferation assay results in mice with PGIA (Fig 2A), individual Cit and R peptide pairs for further testing were selected from the intercept of 2 PG Cit or 2 PG R pools (see peptide P13 as an example in Fig 1A). Since a Cit:R ratio greater than 1.0 was considered as a preferential response to a Cit version of any PG peptide pool, the criteria for selection of individual peptide pairs were as follows: Either both of the intercepting PG peptide pools had to yield Cit:R ratios greater than 1.0, or one of the peptide pools had to yield a Cit:R ratio greater than 1.0 and the other pool had to yield a Cit:R ratio close to (not significantly lower than) 1.0. The following peptide pairs were selected: P1 (at the intercept of PG1 and PG9 pools), P17 (at the intercept of PG3 and PG9 pools), P22 (at the intercept of PG3 and PG14 pools), P33 (at the intercept of PG5 and PG9 pools), P49 (at the intercept of PG7 and PG9 pools), P51 (at the intercept of PG7 and PG11 pools), P54 (at the intercept of PG7 and PG14 pools), and P62 (at the intercept of PG8 and PG14 pools). P57 (at the intercept of PG8 and PG9 pools) was also selected, but both peptides of this pair were insoluble in DMSO or acetonitrile-based solvent and could not be used for any of the assays. The Cit22 peptide also had a solubility problem and was used only in a limited set of assays. It is worth to note that the P51 pair had a substantial sequence overlap with the peptide pair tested by Aggarwal et al [27] in RA patients. Although the PG13 pool consistenly yielded Cit:R ratios much lower than 1.0 in mouse cell proliferation assays (Fig 2A and S1 Fig), the P13 peptide pair was included in the selection for further testing because it could serve as a “negative control” (due to low Cit:R ratio) at least in mice with PGIA [26]. Additionally, the sequences of our P13 pair (S1 Table) overlapped with the peptide pair whose Cit version was found immunogenic in RA patients and healthy control subjects [24][25]. Using spleen cells from mice with PGIA, the selected 9 peptide pairs were then tested for induction of in vitro cell proliferation. As shown in Fig 2B, the peptides (except for P13) yielded Cit:R ratios close to or greater than 1.0. Overall, the preference for Cit peptides in terms of cell proliferation induction was quite modest, with only the P54 pair yielding a Cit:R ratio of significantly greater than 1.0 (*p<0.05).

### Cit:R ratios of soluble cytokines produced by peptide-stimulated spleen cells from mice with PGIA

Although we observed only modest preference for Cit peptides in the proliferative responses of spleen cells from mice with PGIA to the individual PG peptide pairs (Fig 2B), we next tested PGIA spleen cells for preferential release of cytokines in vitro in the presence of Cit peptides versus R peptides. The rationale for proceeding with the cytokine experiments was that Law et al [25] had observed robust cytokine production by human cells in response to a number of citrullinated peptides despite poor proliferative responses to the same peptides. We chose to test the production of pro-inflammatory cytokines IL-17 and IFNγ, as these cytokines can be readily detected in cell cultures from mice with PGIA [30][33][34], and are also relevant to the immunopathology of RA [35][36]. We also tested IL-6, since this pleiotropic pro-inflammatory cytokine is released by cells from mice with PGIA upon in vitro antigen stimulation [33] and IL-6 was also found to be produced by T cells in human PBMC cultures stimulated with citrullinated peptides [25]. Finally, we selected IL-10 for testing in PGIA, as this “anti-inflammatory” cytokine is constitutively secreted by T cells from BALB/c (either naïve or PG-immunized/arthritic) mice [37], and its expression is linked to the regulatory T-cell subset in PGIA and RA [38][39].

**Fig 3:**
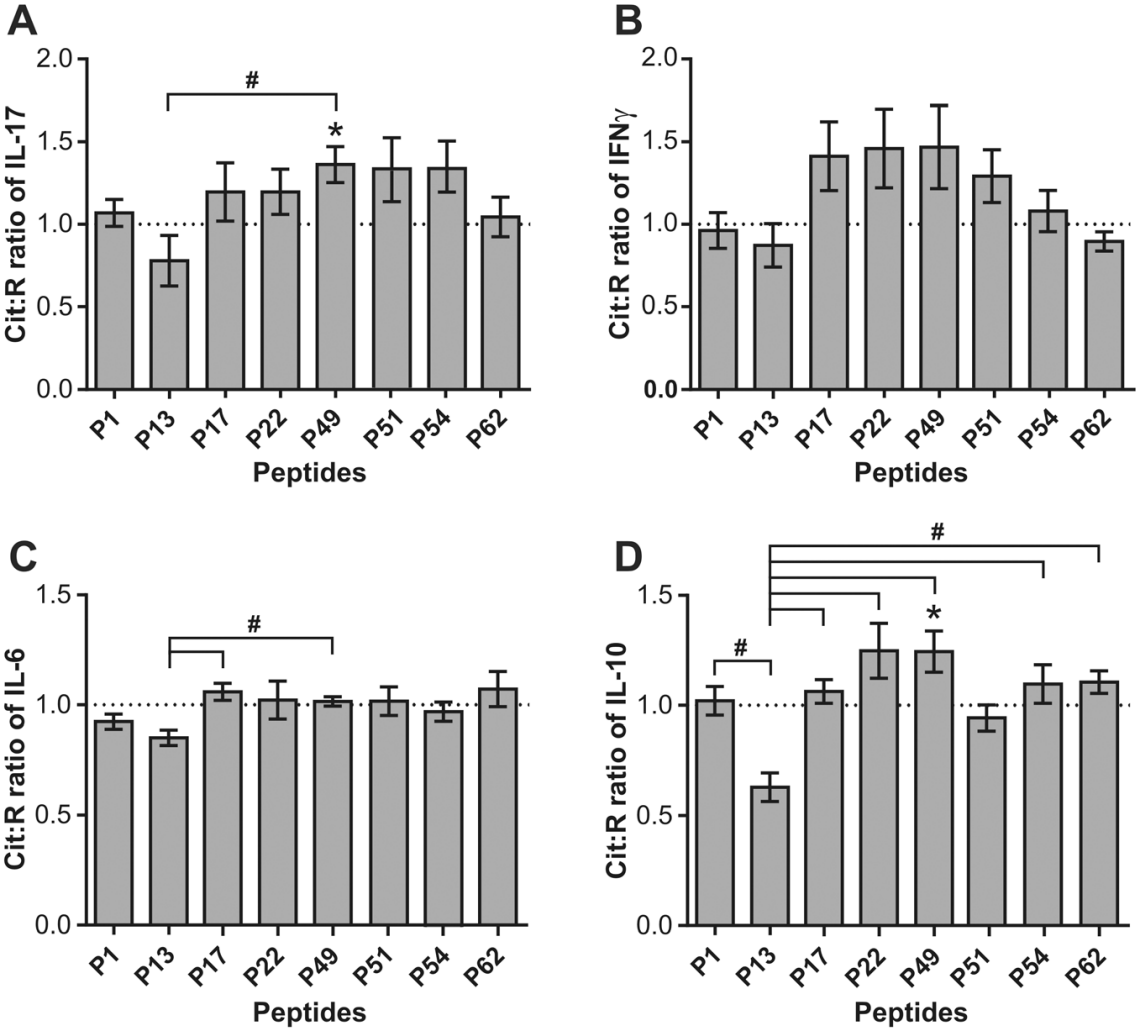
Cit:R ratios of cytokine concentrations in culture supernatants of peptide-stimulated spleen cells from mice with PGIA. Concentrations of cytokines IL-17, IFNγ, IL-6, and IL-10 in 4-day culture supernatants were measured by ELISA. Data are shown as the mean of Cit:R ratios±SEM for the concentrations of (A) IL-17, (B) IFNγ, (C) IL-6, and (D) IL-10. Cit:R ratio of 1.0 is indicated with a dotted line. Wilcoxon signed rank test was used to identify Cit:R ratios significantly higher than 1.0 (preference for a Cit pool or peptide) (*p<0.05; n = 10 mice). Cit:R ratio of the P13 pair-induced vs Cit:R ratio of any other peptide pair-induced production of cytokines in the same cultures was compared using Kruskal-Wallis test followed by Dunn’s multiple comparison test (#p<0.05).

The Cit:R ratio of in vitro IL-17 production (Fig 3A) was significantly greater than 1.0 in the case of P49 (*p<0.05). Five of the 8 peptide pairs (P17, P22, P49, P51, and P54) showed Cit:R ratios greater than 1.0 in eliciting IFNγ (Fig 3B), but none of these values reached significance. The Cit:R ratios of IL-6 production (Fig 3C) were only slightly above 1.0 for P17, P22, P49, P51, and P62. Again, P49 showed a Cit:R ratio significantly greater than 1.0 (*p<0.05) in inducing IL-10 production, although these ratios were slightly above 1.0 for P17, P22, P54 and P62 as well (Fig 3D). As expected, the Cit:R ratio of the P13 pair was found to be the lowest in all of the four cytokine assays, and the Cit:R ratios of IL-17, IL-6, and IL-10 were significantly higher for P49 and a few other peptide pairs than the Cit:R ratio of P13 (#p<0.05) (Fig 3A, 3C and 3D). In summary, the P49 pair stood out by showing clear preference (significantly elevated Cit:R ratio) for peptide Cit49 in the production of two of the four cytokines tested (Fig 3A and 3D), and the Cit:R ratio of the P49 pair was significantly higher (#p<0.05) than the Cit:R ratio of the negative control P13 pair in three of the four cytokine assays (Fig 3A, 3C and 3D).

### Comparison of spleen cells from naïve mice and mice with PGIA or GIA for cytokine production in response to the P13 and P49 peptide pairs

**Fig 4:**
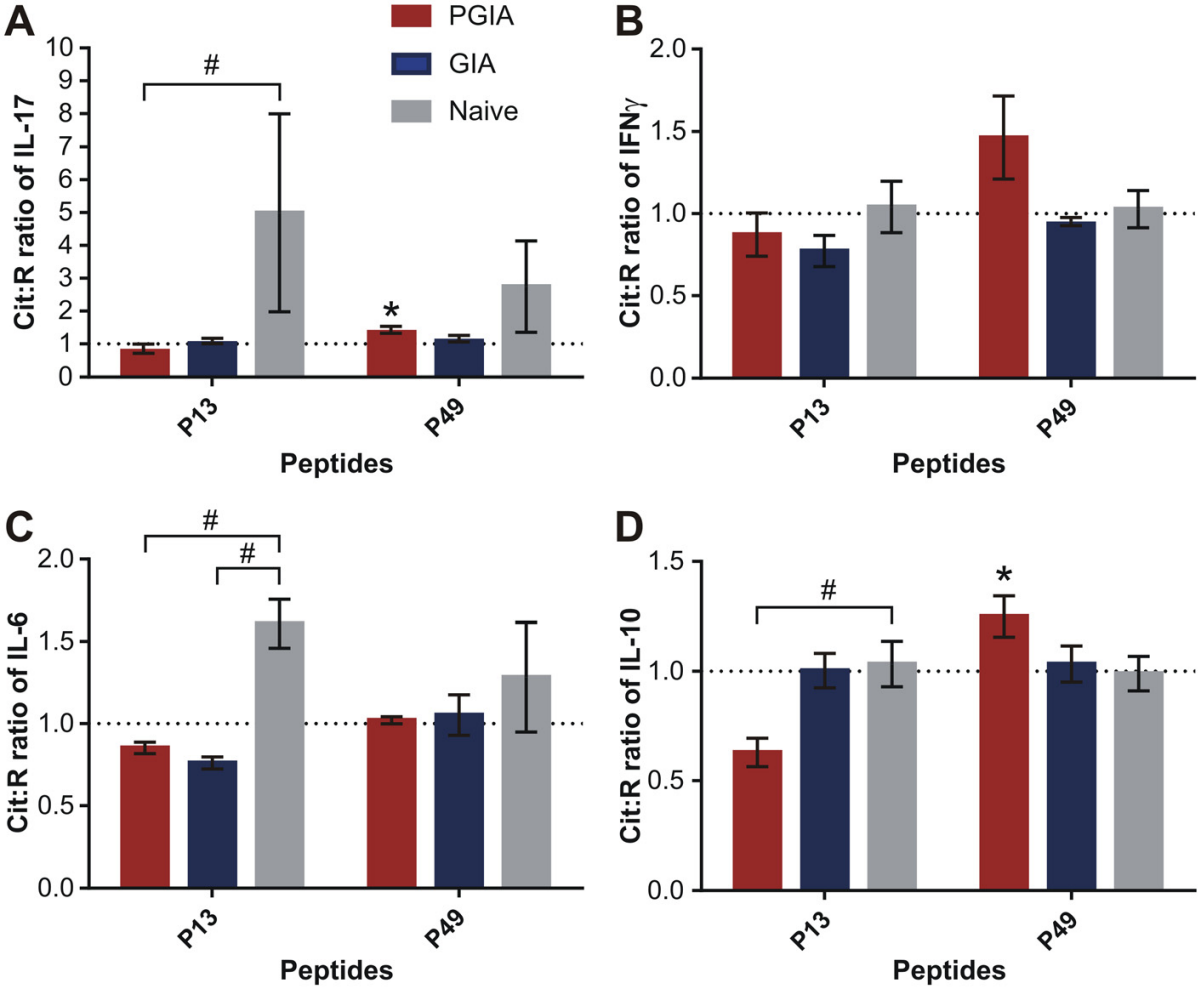
Cit:R ratios of IL-17, IFNγ, IL-6, and IL-10 concentrations in peptide-stimulated culture supernatants of spleen cells from mice with PGIA or G1 domain-induced arthritis (GIA) or from naïve mice. Data are shown as the mean of Cit:R ratios±SEM for the concentrations of (A) IL-17, (B), IFNγ, (C) IL-6, and (D) IL-10 in peptide-treated spleen cell cultures from mice with PGIA (red bars) or GIA (blue bars) or from non-arthritic naïve mice (gray bars) (PGIA n = 10 mice; GIA n = 5 mice; Naïve n = 3 mice). Cit:R ratio of 1.0 is indicated by a dotted line in each graph. Wilcoxon signed rank test was used to identify Cit:R ratios significantly higher than 1.0 (*p = <0.05: Cit:R ratio vs 1.0). Cit:R ratios of cytokines induced by the P13 and P49 pairs of peptides in cells from the 3 groups of mice were compared using Kruskal-Wallis test followed by Dunn’s multiple comparison test (#p<0.05: PGIA or GIA vs naïve mice). Average net concentrations of IL-17 in the cell cultures stimulated with the Cit version of P13 (Cit13) were as follows: 86.4 pg/ml in the PGIA group, 70.5 pg/ml in the GIA group, and 76.8 pg/ml in the naïve group (graphs not shown). In the Cit49-stimulated cultures, the average net amounts of IL-17 were: 136.9 pg/ml in the PGIA group, 60.8 pg/ml in the GIA group, and 29.8 pg/ml in the naïve group (graphs not shown).

We next asked whether the poor overall cytokine responses to the Cit version of P13 and the preference for the Cit version of P49 in the production of IL-17 and IL-10 (Fig 3) was specific for the PGIA model or also occurred in GIA or in naïve BALB/c mice. Surprisingly, when spleen cells from these 3 groups of mice were tested for in vitro IL-17 production (Fig 4A), naïve mice showed much greater preference for Cit13 (#p<0.05) than the cells from mice with either PGIA or GIA. Despite these preferences exhibited by naïve cells, the net amounts of IL-17 were similar in the Cit13-stimulated cultures of the 3 groups of mice (Fig 4 legend). Furthermore, despite having no significant differences among the groups in the Cit:R ratios of P49-stimulated IL-17 secretion (Fig 4A), the net amount of IL-17 was highest in the Cit49-stimulated cultures of PGIA mice (Fig 4 legend). Cells from mice with PGIA showed slightly elevated IFNγ production in response to the Cit version as compared to the R version of P49 (Fig 4B). The Cit:R ratios of IL-6 (Fig 4C) and IL-10 (Fig 4D) in response to P13 were also higher in naïve mice than in those with PGIA or GIA. Cells from mice with GIA did not exhibit preferential responses to either Cit13 or Cit49 in the production of any of the four cytokines (Fig 4). In summary, these results suggested that preferential recognition of the Cit13 peptide (in terms of IL-17 and IL-6 production) existed in naïve mice, but such preference was not observed in rhG1- or PG-immunized arthritic mice. With regard to the net concentrations of IL-17, preference for the Cit version of the P13 peptide by naïve cells was not necessarily associated with elevated amounts of this cytokine in the Cit13-stimulated cultures of naïve cells as compared to GIA or PGIA cells (Fig 4 legend). Overall, mice with PGIA responded more preferentially to Cit49 than the other groups, as the Cit:R ratios of cytokines elicited by P49 were greater than 1.0 (although did not always reach significance) in PGIA cell cultures. Cells from mice with GIA did not show preference at all for the Cit version over the R version of either P13 or P49.

### Serum anti-Cit peptide Abs and Cit:R Ab ratios in mice with PGIA or GIA, and in naïve mice

**Fig 5:**
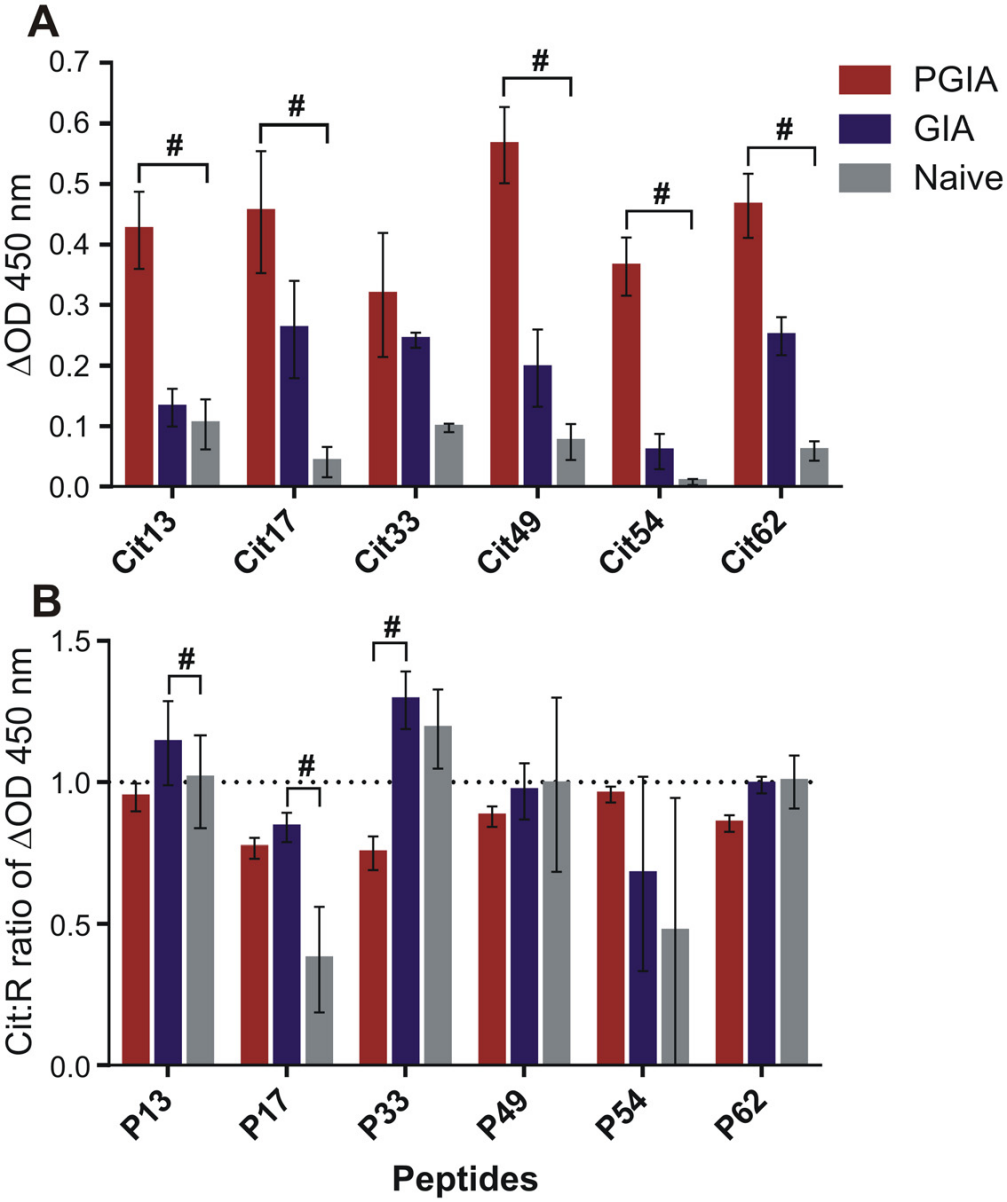
Anti-PG peptide antibodies in serum samples from mice with PGIA or GIA, or from naïve mice. IgG antibodies reacting with Cit or R versions of 6 PG peptides were measured by ELISA using biotinylated Cit-R peptide pairs. Delta optical density at 450 nm (ΔOD 450 nm) was determined as described in the Methods. (A) Serum antibodies against the Cit versions of six PG peptides in mice with PGIA (red bars) or GIA (blue bars) or naïve mice (gray bars). Data are shown as the mean ΔOD 450 nm±SEM (PGIA n = 10 mice/peptide; GIA n = 3–5 mice/peptide; Naïve n = 3 mice/peptide). Inter-group comparisons were made using Kruskal-Wallis test followed by Dunn’s multiple comparison test (^#^p<0.05: PGIA vs naïve mice). (B) Cit:R ratios of peptide-specific serum antibodies from mice with PGIA or GIA and from naïve mice. Data are expressed as Cit:R ratios of ΔOD 450 nm (mean±SEM) of serum antibodies specific for the peptide pairs in the groups of mice described above. Inter-group comparisons were made as described for graph A above (#p<0.05: GIA vs naïve or PGIA vs GIA mice). A Cit:R ratio of 1.0 is indicated by a dotted line. None of the Cit:R ratios were significantly different from 1.0 as determined by Wilcoxon signed rank test.

We sought to determine if mice with PGIA or GIA and unimmunized naïve mice produced Abs to Cit PG peptides and whether the Cit peptide preference pattern of serum Abs reflected the preference profile of T-cell cytokine secretion in these groups of mice. Since the Cit:R ratios of secreted IL-17 and IL-10 were significantly elevated in P49-treated cells from mice with PGIA (Fig 3A and 3D), and IL-17 [40] as well as IL-10 [41] can promote Ab production, we anticipated high serum levels of Abs against Cit49 or other Cit peptides in the PGIA group. As indicated by the ΔOD values of anti-PG peptide Ab assays, the serum of mice with PGIA contained considerable amounts of Abs (IgG) against the Cit versions of all of the 6 peptides tested (P13, P17, P33, P49, P54, and P62) with serum anti-Cit49 levels being the highest in this group (Fig 5A). Ab levels against these peptides were lower in mice with GIA than in those with PGIA, and anti-Cit peptide Abs were barely detectable in the serum of naïve mice (#p<0.05, PGIA vs naïve) (Fig 5A). Irrespective of the amounts of anti-peptide Abs, Cit over R preference was not observed in the sera of PGIA or naïve mice (Cit:R ratios did not exceed 1.0 significantly) (Fig 5B). These results suggested that although the sera of mice with PGIA and GIA contained well-detectable amounts of Abs against multiple Cit peptides, significant preference for the Cit over the R forms of these peptides was not observed. In other words, preferential T-cell recognition of some citrullinated epitopes of PG was not accompanied by a preference of Ab (B-cell) recognition of Cit peptides in any of the three groups of mice tested in this study.

### Cit:R ratios of cytokine release in response to pooled PG peptide stimulation of PBMC from a RA patient and a healthy control subject

**Fig 6:**
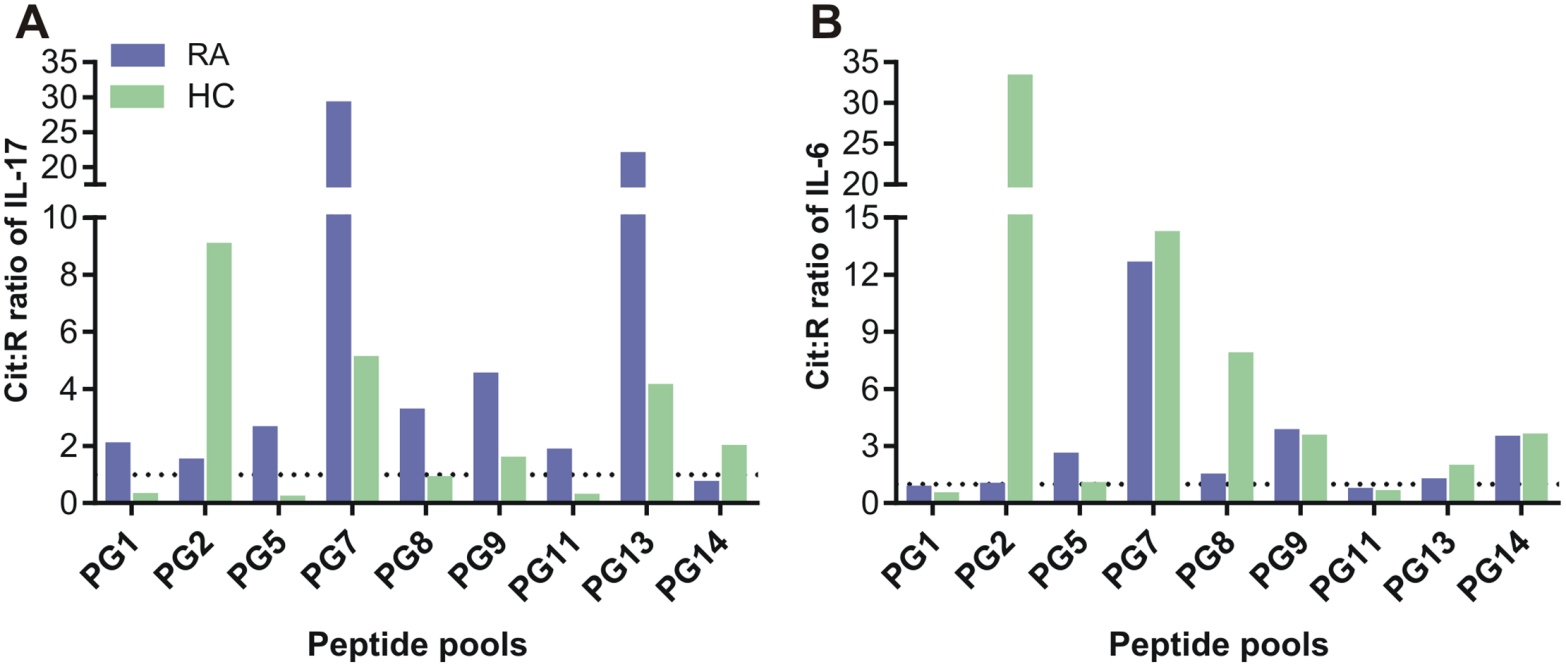
Cit:R ratios of PG peptide pool-induced IL-17 and IL-6 concentrations in supernatans of human peripheral blood mononuclear cell (PBMC) cultures. (A) IL-17 and (B) IL-6 concentrations in PG peptide pool-stimulated PBMC cultures of a patient with rheumatoid arthritis (RA, blue bars) and a healthy control (HC, green bars) subject were measured by ELISA as described in the Methods. Data are expressed as the mean Cit:R ratios of the indicated cytokines induced in RA and HC PBMC by Cit and corresponding R versions of 9 PG peptide pools. Cit:R ratio of 1.0 is depicted by a dotted line.

We obtained large numbers of PBMC (>6x10^7^ cells) from one RA patient (ACPA-) and a healthy control (HC) subject, which allowed us to test IL-17 and IL-6 release upon culture with 9 of the 16 pairs of the PG peptide pools as well as with individual peptide pairs. In order to assess if, similarly to mice, the responses of human cells could predict a trend of preferential recognition of Cit peptides positioned at the intercept of preferred Cit PG peptide pools (see Figs 1A and 2A), we chose 7 intercepting PG pools that scored relatively high (Cit:R ratio: ≥1.0) and 2 pools that scored low (Cit:R ratio: <1.0) in the proliferation assays of PGIA splenocytes (see Fig 2A) for testing with human PBMC. With regard to IL-17 production by peptide pool-stimulated cells, PBMC from the RA patient showed strong preference for the Cit versions of the PG7, PG9, and PG13 pools, while the HC cells preferred the Cit pools of PG2, PG7, and PG13 (Fig 6A). The Cit:R ratios of IL-6 production by the RA PBMC were the highest in the case of the PG7 pool, while HC cells preferentially released IL-6 in the presence of Cit pools PG2, PG7, and PG8 (Fig 6B). As the Cit pools of PG7 and PG9 contained the Cit49 peptide, and the PG2 and PG13 Cit pools had the Cit13 peptide, these results suggested that preferential IL-17 production of RA cells in response to Cit49 could be similar to that of mice with PGIA (see Fig 3A), whereas IL-17 release by HC cells in favor of Cit13 could be similar to the preference of naïve mouse cells for the same peptide (see Fig 4A). This observation prompted us to focus on the cytokine responses to the Cit-R pairs of P13 and P49 by PMBCs from RA patients and HC individuals, although a few other PG peptide pairs were also tested in cell cultures of a limited number of human subjects.

### IL-17, IFNγ, and IL-6 production by RA and HC PMBCs in response to Cit and R forms of PG peptide pairs

**Fig 7:**
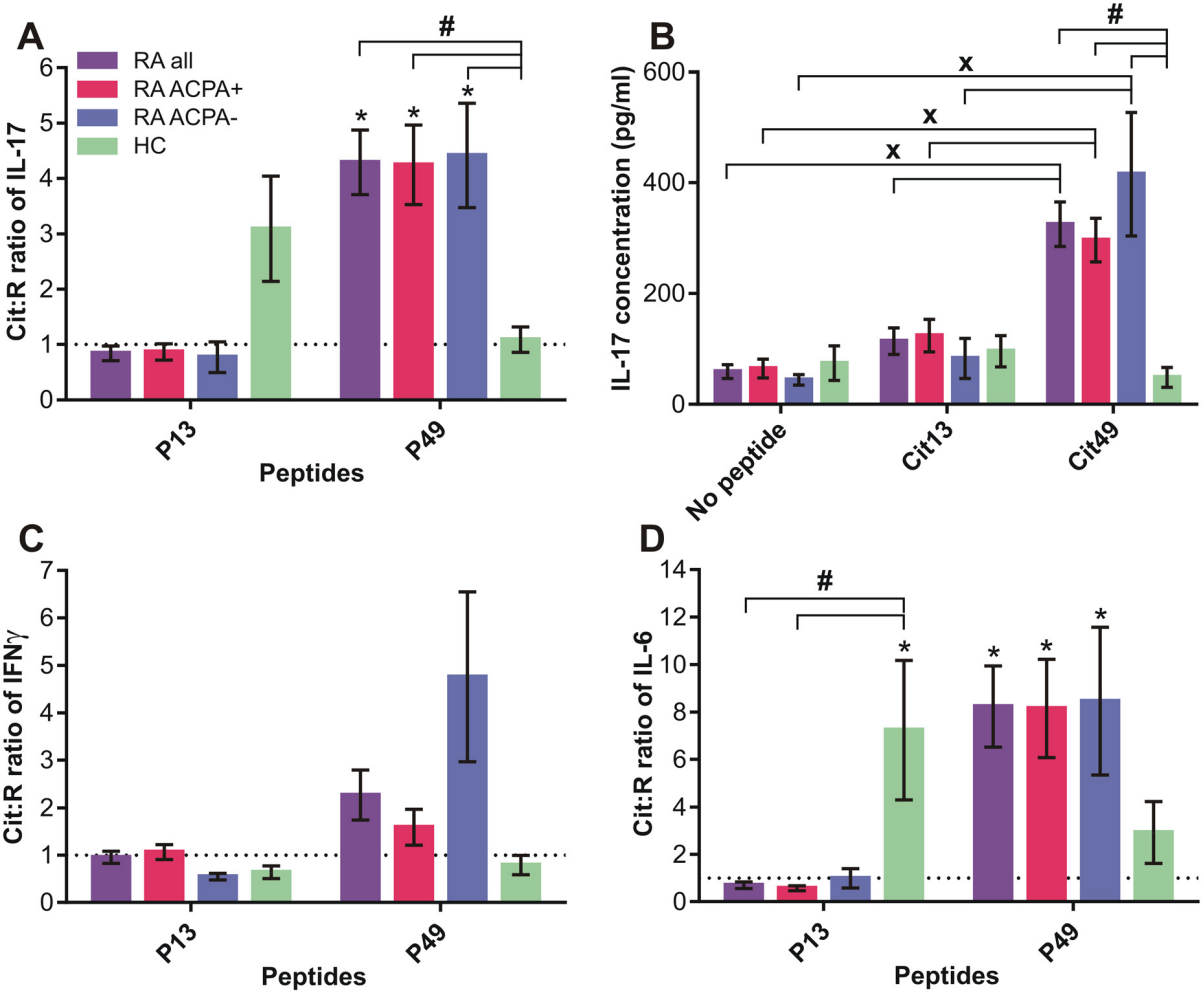
Cit:R ratios of IL-17, IFNγ, and IL-6, induced in human PBMC cultures by Cit and R pairs of P13 and P49. (A) Cit:R ratios of IL-17 induced by the P13 and P49 peptide pairs in PBMC cultures of anti-citrullinated protein antibody positive (ACPA+) and ACPA negative (ACPA-) RA patients and HC subjects. Data are expressed as the mean±SEM of Cit:R ratios of peptide-induced IL-17 in PBMC of RA all (purple bars), RA ACPA+ (red bars), RA ACPA- (blue bars) and HC (green bars) groups. (B) Net concentrations of IL-17 in the same PBMC cultures produced in the absence of a peptide (no peptide) or in the presence of peptide Cit13 or Cit49. Data shown are the mean±SEM of IL-17 (pg/ml). Cit:R ratios of (C) IFNγ and (D) IL-6 in the P13 and P49 peptide pair-stimulated PBMC cultures were determined as described for IL-17 in panel A above. Sample numbers for IL-17 (RA all n = 42; RA ACPA+ n = 32; RA ACPA- n = 10; HC n = 8), for IFNγ (RA all n = 28; RA ACPA+ n = 22; RA ACPA- n = 6; HC n = 8), and for IL-6 (RA all n = 41; RA ACPA+ n = 31; RA ACPA- n = 10; HC n = 7). Cit:R ratio of 1.0 (in panels A, C and D) is indicated by a dotted line. Cit:R ratios of cytokines (in A, C and D) were analyzed using Wilcoxon signed rank test (*p<0.05: Cit:R ratio vs 1.0) and Kruskal-Wallis test followed by Dunn’s multiple comparison test (#p<0.05: RA groups vs HC group). For the data in panel B, inter-group and inter-peptide comparisons were made using two-way ANOVA followed by Tukey’s test (#p<0.05: RA groups vs HC group) and Shidak’s test (^X^p<0.05: Cit49 vs Cit13 or no peptide).

Although we collected plasma from all of the 46 RA patients and 9 HC subjects, the PBMC yield from 4 patients and 1 HC was insufficient to perform any cell-based assay. We had enough PBMC from 42 RA and 8 HC subjects to test cytokine release in response to in vitro stimulation with the P13 and P49 peptide pairs, but fewer blood samples could be tested for cell responses to additional PG peptide pairs. When we cultured PBMC from the 42 RA patients (32 ACPA+ and 10 ACPA-) and 8 HC subjects in the absence or presence of Cit and R versions of P13 and P49, we detected preferential IL-17 production by HC cells in the presence of the Cit13 peptide as compared to the R13 peptide, and a strong preference of cells from all RA patients (both ACPA+ and ACPA-) for Cit49 (*p<0.05: Cit:R ratios vs 1.0) (Fig 7A). These human IL-17 results were reminiscent of those obtained from peptide-treated spleen cell cultures of naïve mice and of mice with PGIA, respectively (see Fig 4A). However, as observed in mice (Fig 4 legend), the concentrations of IL-17 in the cultures containing no peptide, or Cit13 or Cit49 peptide, were significantly elevated only in RA (both ACPA+ and ACPA-) cultures in the case of Cit49 (#p<0.05: RA vs HC; ^X^p<0.05: Cit49 vs Cit13 or no peptide) (Fig 7B), suggesting that the Cit49 peptide elicited robust IL-17 production only from RA cells. With regard to IFNγ production, cells from ACPA- RA patients showed a trend of preferential response to Cit49, but this did not reach significance (Fig 7C). The Cit:R ratios of IL-6 production in response to the P13 and P49 peptides were similar to those of IL-17 (*p<0.05: Cit:R ratios vs 1.0) (compare Fig 7D and 7A). Of note, the amounts of supernatant were insufficient in some of the PBMC cultures for measurement of IFNγ or IL-6 (sample numbers are given in the legend of Fig 7).

Production of IL-17, IL-6, and IFNγ by PBMC was also determined after stimulation with the Cit and R forms of peptides P17, P33, P51, P54, and P62 (S2 Fig). Cytokine responses to these peptides varied widely; a Cit:R ratio significantly higher than 1.0 was found only in the case of IL-6 release from HC PBMC in response to P33 stimulation (*p<0.05), and this ratio was also significantly greater in cell cultures of HC subjects than those of ACPA+ RA patients (#p<0.05) (S2C Fig). Due to the high variance of cytokine secretion in response to these peptides and the relatively low number of human samples tested, the statistical power was insufficient to establish significant preference for the Cit versions of P17, P51, P54, or P62 in these human cell cultures.

Simultaneous production of IL-17 and IL-6 by the Cit49 peptide-treated RA PBMC cultures (Fig 7A, 7B and 7D) is of particular interest, as these two cytokines are known to be involved in the pathogenesis of RA [35]. IL-17 and IL-6 both contribute to joint tissue destruction directly by upregulating pro-inflammatory pathways and enhancing bone resorption [42][43] as well as indirectly by promoting autoAb production [42][44]. RA articular cartilage, which may contain citrullinated PG [23], can serve as a reservoir of Cit PG epitopes potentially triggering local IL-17 and IL-6 production by joint-infiltrating T cells, although the Cit49 epitope in RA cartilage remains to be identified.

### Intracellular IL-17A and IFNγ contents of CD4+ cells in Cit or R peptide-stimulated PBMC cultures of RA patients and HC subjects

**Fig 8:**
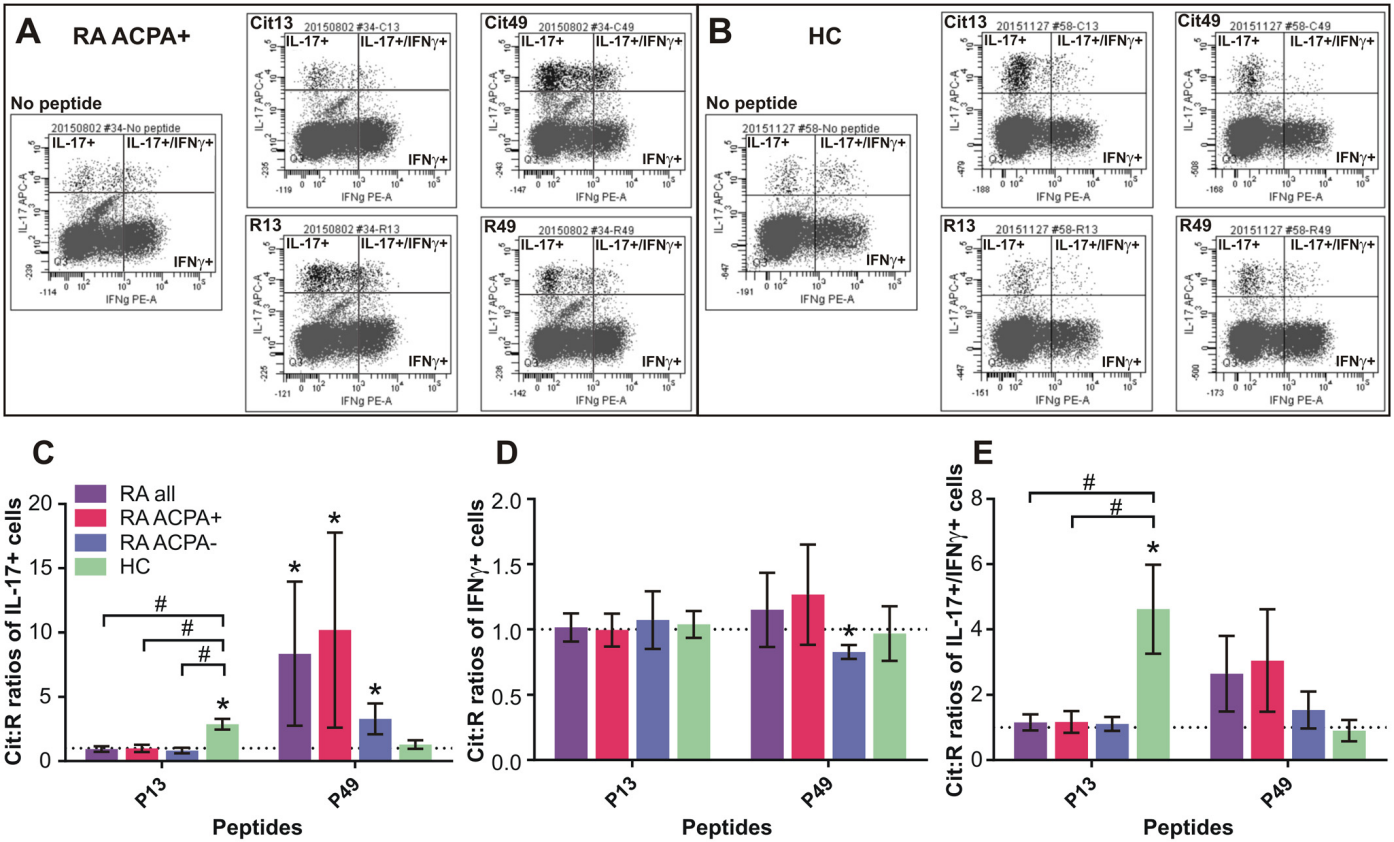
Intracellular cytokines in CD4 cells from non-stimulated or peptide-stimulated PBMC cultures of RA patients and HC subjects. Representative flow cytometry panels of intracellular cytokines in CD4+ cells from non-stimulated or peptide-stimulated PBMC cultures of (A) an ACPA+ RA patient and (B) a HC subject. PBMC were cultured in the absence of peptide (no peptide) or in the presence of Cit or R versions of P13 (Cit13, R13) or P49 (Cit49, R49). Cells were labeled for cell-surface CD4 and intracellular IL-17 (IL-17A) and IFNγ with fluorochrome-tagged antibodies and subjected to flow cytometry as described in the Methods. In each case, the gate was set at CD4+ cells (gate not shown) and fluorescence signals were collected in 2-dimensional dot plots. Dots in the upper left quadrants of flow panels represent CD4 cells containing IL-17, dots in the lower right quadrants are cells containing IFNγ, the upper right quadrants show cells containing both IL-17 and IFNγ, and the cells in the lower left quadrants do not contain either of these cytokines. Cit:R ratios of (C) IL-17+ cells (D) IFNγ+ cells, and (E) double IL-17/IFNγ+ cells in P13 or P49-stimulated PBMC cultures of RA and HC groups. Data are expressed as mean±SEM of Cit:R ratios of cytokine-containing cells. Sample numbers for intracellular IL-17A, IFNγ, and IL-17/IFNγ (RA all n = 34; RA ACPA+ n = 23; RA ACPA- n = 9; HC n = 8). Cit:R ratio of 1.0 is indicated by a dotted line. Cit:R ratios significantly higher or lower than 1.0 were identified by Wilcoxon signed rank test (*p<0.05: Cit:R ratio vs 1.0) and Cit:R ratios of the groups were compared using Kruskal-Wallis test followed by Dunn’s multiple comparison test (#p<0.05: RA groups vs HC group).

To corroborate the results of soluble cytokine production by the PG peptide-stimulated PBMC, and to determine T helper (Th) cell polarization, we performed intracellular staining of CD4+ cells from the same cultures for IL-17A and IFNγ content in all cases where the cell yield was sufficient for flow cytometric analysis. Fig 8A and 8B show examples of flow cytometry profiles of intracellular cytokines in CD4-gated cells from an ACPA+ RA patient and a HC subject, respectively. As seen in Fig 8A and 8B, both the RA patient and the HC had remarkable proportions of IFNy+ CD4 cells (Th1) (lower right quadrants) and detectable numbers of IL-17A+ CD4 cells (Th17) (upper left quadrants) even in the absence of any peptide (No peptide). Interestingly, smaller, but still well detectable, populations of dual IL-17A+/IFNγ+ (Th17/Th1) CD4 cells were also present (upper right quadrants). As compared to baseline (Fig 8A, left flow panel: No peptide), stimulation with the Cit13 peptide did not induce increases in the proportions of Th17 or Th17/Th1 cells in the RA culture (upper middle panel), but the R13 peptide did (lower middle panel). In contrast, the Cit49 peptide induced a robust expansion of both Th17 and Th17/Th1 cells (upper right panel), while R49 did not (lower right panel). HC cells showed the opposite effects (Fig 8B): Cit13 was a stronger inducer of Th17 and Th17/Th1 cells than R13 (middle panels), and Cit49 was a weaker inducer of Th17/Th1 cells than the R49 peptide (right panels) in HC cultures. As exemplified in Fig 8A and 8B, the IFNγ+ Th1 cell populations were quite similar in size in RA and HC cultures and did not change much in response to stimulation with any of the peptides, but the Th17 and Th17/Th1 populations were generally smaller in HC than in RA cultures and showed peptide-specific changes as described above. When the Cit:R ratios of cytokine positive cells were calculated (Fig 8C–8E), we found that, indeed, Cit13-stimulated HC cultures contained a significantly higher proportion of Th17 cells than R13-treated cultures, but RA cultures (especially those from ACPA+ patients) yielded significantly more Th17 cells than HC cultures in response to the Cit version of P49 (*p<0.05, Cit:R ratios vs 1.0) (Fig 8C). However, the sizes of the IFNγ-containing Th1 populations were similar in Cit13- and R13-stimulated cultures of RA and HC cells, and ACPA- RA cultures yielded even lower proportions of Th1 cells in Cit49-treated than in R49-treated cultures (*p<0.05) (Fig 8D). Similarly to Th17 cells, Th17/Th1 cells in HC cultures demonstrated a striking preference for the Cit version of P13 as compared with RA cells (*p<0.05: Cit:R ratios vs 1.0) (Fig 8E). The Th17/Th1 populations in RA cultures showed a trend of expansion in favor of the Cit49 peptide, but this did not reach significance (Fig 8E).

Cit and R pairs of P17, P33, P51, P54 and P62 peptides were also tested for differential effects on intracellular cytokine-containing CD4 cells in a limited number of PBMC cultures. As in the case of secreted cytokines, stimulation with these peptide pairs yielded highly varying Cit:R ratios of Th17, Th1 and Th17/Th1 cells, but none of these ratios was significantly higher than 1.0 (S3 Fig). The Cit:R ratio of IFNγ-containing Th1 cells in the P17-treated cultures of ACPA+ RA patients was even significantly lower than 1.0 (*p<0.05) and was also lower than the Cit:R ratio of IFNγ in P17-treated HC cultures (#p<0.05: RA vs HC) (S3B Fig). Overall, the results of intracellular IL-17A and IFNγ measurements corroborated the results of soluble cytokines released by the same PBMC cultures.

An interesting aspect of our intracellular cytokine studies was the detection of a remarkable population of human Th17/Th1 cells co-expressing IL-17A and IFNγ. Although expansion of T cells by treatment with the homeostatic cytokine IL-2 could, at least in part, account for the enrichment of the cultures in a dual Th17/Th1 subset, in vivo existence of Th17/Th1 cells in the gut of patients with Crohn’s disease [45] and in the synovial fluid of juvenile idiopathic arthritis patients [46] has been demonstrated. Generation of Th17/Th1 clones from normal PBMC by in vitro treatment with IL-12 has also been reported [45]. Acquisition of IFNγ production by Th17 cells is a prominent feature of phenotypic and functional plasticity of Th subsets, and dual Th17/Th1 cells are thought to arise under inflammatory conditions and to represent a pathogenic population in autoimmune diseases [46][47].

### Anti-Cit PG peptide Abs in the plasma of RA patients and HC subjects

**Fig 9:**
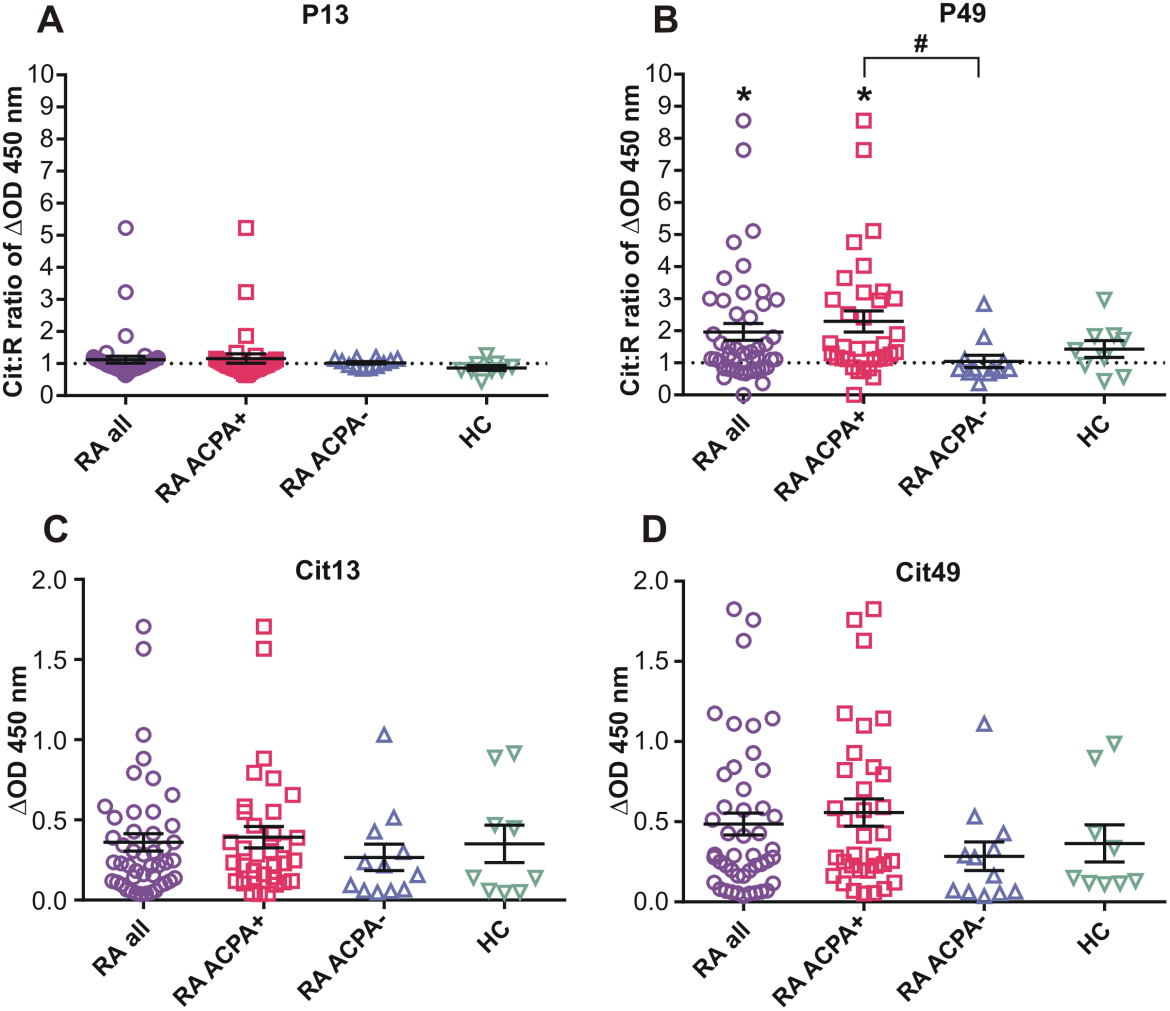
Antibodies reacting with P13 and P49 peptides in plasma samples from RA patients and HC subjects. IgG antibodies reacting with Cit or R versions of P13 and P49 in the human plasma samples were detected by ELISA as described in the Methods. Cit:R ratios of (A) anti-P13 and (B) anti-P49 antibodies in plasma samples from RA and HC subjects. ΔOD 450 nm values representing plasma levels of (C) anti-Cit13 and (D) anti-Cit49 antibodies. Data shown are the mean±SEM. Sample numbers for both peptide pairs (RA all n = 46; RA ACPA+ n = 34; RA ACPA- n = 12; HC n = 9). Cit:R ratio of 1.0 is indicated by a dotted line. Statistical analysis was performed using Wilcoxon signed rank test (*p<0.05: Cit:R ratio vs 1.0) and Kruskal-Wallis test followed by Dunn’s multiple comparison test (#p<0.05: ACPA+ group vs ACPA- group).

Finally, we asked whether preferential T-cell recognition of the Cit forms of P13 and P49 (and other) peptides was accompanied by the presence of Abs (IgG) against the Cit versions of these peptides in the plasma of RA patients or HC subjects. The Cit:R ratios of ΔOD values obtained from anti-PG peptide ELISA assays using a biotinylated Cit and R pair of P13 were sligthly elevated in the plasma of only 3 of 46 RA patients; all these samples came from the ACPA+ group (Fig 9A, compare RA all and ACPA+). Abs from ACPA- RA patients or HC subjects did not exhibit preference for the Cit version of P13 (Fig 9A). In sharp contrast, the Cit:R ratios of anti-P49 Abs were significantly elevated (*p<0.05, Cit:R ratios vs 1.0) in the plasma of RA patients, and, again, nearly all of these samples came from the ACPA+ group (Fig 9B). There was no preference for the Cit49 peptide in the ACPA- RA and HC groups, and, in fact, the Cit:R ratios of anti-P49 Abs were significantly lower in ACPA- than in ACPA+ RA plasma samples (#p<0.05: ACPA- vs ACPA+) (Fig 9B). Comparison of plasma levels (indicated by ΔOD) of Abs against Cit13 or Cit49 did not reveal significant differences among the groups tested, although the average level of anti-Cit49 Abs was the highest in ACPA+ RA patients (Fig 9C and 9D).

Testing of Cit and R pairs of P17, P33, P54, and P62 revealed no significant preference of Ab reactivity with the Cit forms of these peptides in either the RA groups or the HC group, but the Cit:R ratios of Abs against each of these 4 peptides were slightly higher than 1.0 in a few ACPA+ and ACPA- RA plasma samples (S4 Fig).

### T-cell and Ab (B-cell) recognition of the Cit49 epitope in ACPA+ RA patients

**Fig 10:**
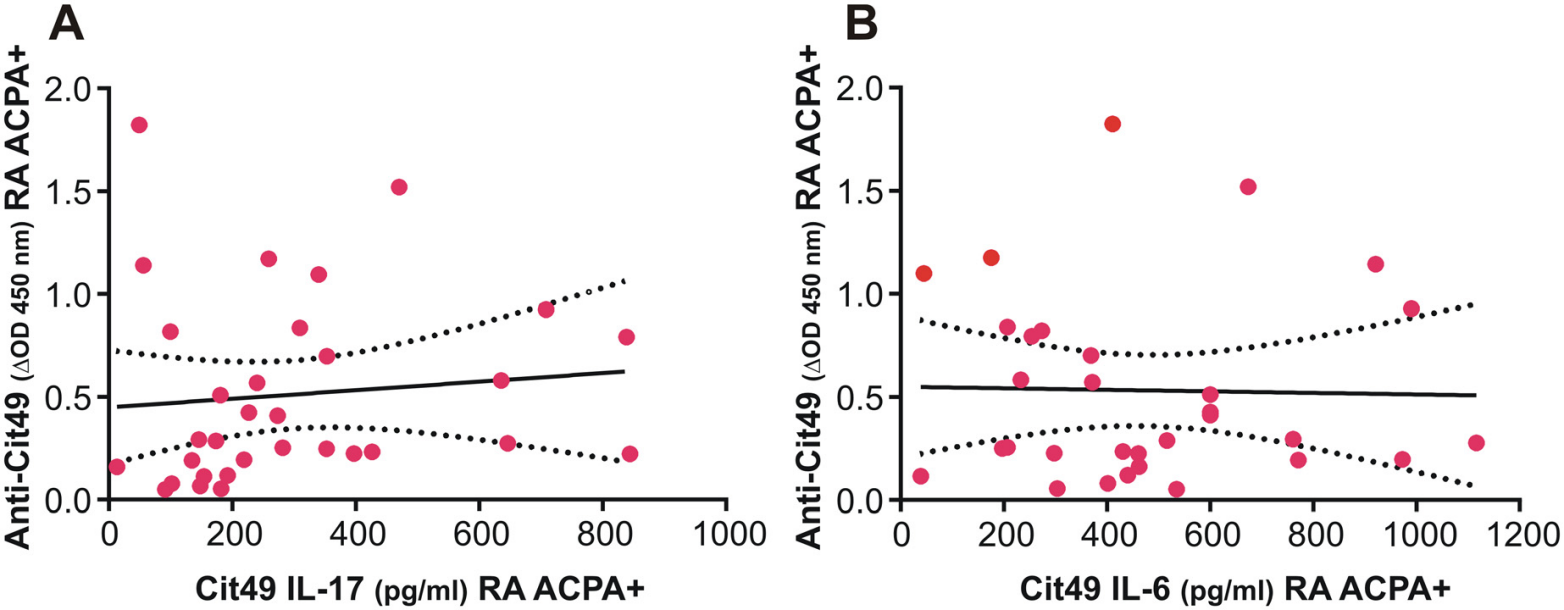
Correlation analysis of plasma anti-Cit49 Ab levels and in vitro Cit49-specific cytokine production in ACPA+ RA patients. (A) Analysis of correlation between anti-Cit Ab levels (ΔOD 450 nm) and Cit49-induced IL-17 (pg/ml) in PBMC culture supernatants of the RA ACPA+ group. No significant correlation was found between these parameters (r = 0.1022, R^2^ = 0.0105, p = 0.5777; n = 32). (B) Analysis of correlation between anti-Cit Ab levels (ΔOD 450 nm) and Cit49-induced IL-6 (pg/ml) in PBMC culture supernatants of the RA ACPA+ group revealed no significant correlation (r = -0.0225, R^2^ = 0.0005, p = 0.9043; n = 31). Analyses were carried out using Pearson’s correlation test and best-fit curves (black solid lines) were obtained through linear regression. Sample values are shown as red dots. The black dotted lines represent 95% confidence intervals.

Overall, the results of anti-PG peptide Ab assays suggested co-existence of preferential recognition of the Cit form of P49 by both CD4+ T cells (Th17 and Th17/Th1 subsets) and IgG Abs, predominantly in ACPA+ RA patients. T-cell preference for Cit49 was found in ACPA- RA patients and for Cit13 in HC subjects (Figs 7 and 8). However, preferential Ab (IgG) reactivity with these Cit peptides was not detected in either of these two groups (Fig 9). Furthermore, high amounts of IL-17 were detected in the supernatants of Cit49-stimulated cells from both ACPA+ and ACPA- RA patients (Fig 7B), yet the plasma levels of anti-Cit49 Abs were higher in the ACPA+ than the ACPA- group (Fig 9B and 9D). The latter observations suggested that recognition of the Cit49 epitope by T cells could be linked to an already existing humoral response to this epitope in ACPA+ patients. However, correlation analysis of Cit49-induced cytokine (IL-17 and IL-6) production and anti-Cit49-specific Ab levels in the ACPA+ RA group revelad no significant association between these parameters (Fig 10). Low-to-moderate levels of cytokines (induced in vitro by Cit49) and anti-Cit49 Abs were found to co-exist in the majority of these patients, while some ACPA+ RA patients with the highest levels of anti-Cit49 Abs had very low T-cell reactivity (in terms of IL-17 or IL-6 production) with this peptide (Fig 10). These observations suggest that T-cell recognition of, and Ab reactivity with, the Cit49 epitope are not dependent on each other. Recognition of distinct epitopes by T cells and Abs within the same multi-determinant antigen is a long-held view [48]. On the other hand, expansion of the ACPA repertoire via intra- and intermolecular epitope spreading before and after the development of RA is a well-demonstrated phenomenon [5][8]. It is possible, therefore, that ACPA reacting with the Cit49 epitope arises through epitope spreading after an initial autoimmune reaction with another citrullinated epitope in the PG molecule or even with a citrullinated epitope in a different protein.

### Similarities and differences between mice and humans in the immune recognition pattern of PG epitopes

We used BALB/c mice with PGIA as a screening tool to help pre-select preferentially recognized Cit PG peptides for further testing in humans. Since these mice were immunized with human OA PG that likely contained both R and Cit epitopes [23], it is not surprising that their T and B cells discriminated relatively poorly between the R and Cit forms of the PG peptides (Figs 2–4). It is important to note that the human P13 peptide (ATEGRVRVNSAYQDK) (S1 Table) has only 86% homology with the mouse sequence (ATEGQVRVNSIYQDK, different aa residues are underlined; see mouse PG full sequence at http://www.uniprot.org/uniprot/Q61282#sequences). Therefore, the immunodominant nature of the R13 peptide in mice with PGIA might, at least in part, be related to its recognition as a foreign epitope, and substitution of the first R residue (Q in mouse) with Cit in the human sequence might even reduce the overall immunogenicity of the peptide in mice. In contrast, the human P49 peptide (MDMCSAGWLADRSVR) (S1 Table) is 100% homologous with the mouse sequence, with only its Cit version being potentially recognized as “altered self” in mice. Although the degree of discrimination between the Cit and R forms of P13 and P49 was greater in humans than in mice, there were remarkable similarities between the two species. Th17 cells from naïve mice and human HC subjects reacted more strongly with the Cit than the R version of P13, while Th17 cells from mice with PGIA and from humans with RA both showed preference for the Cit form over the R form of P49 (Figs 4A, 7A and 8C). In addition, the levels of circulating Abs against Cit49 were the highest in mice with PGIA and in patients with ACPA+ RA (Figs 5A and 9D). In the case of GIA (arthritic mice immunized with non-citrullinated rhG1 protein), the similarity between the GIA model and patients with ACPA- RA was not clear and was limited to the detection of relatively low levels of circulating anti-Cit PG peptide Abs in the respective groups (Figs 5A and 9D). Overall, the results of our study demonstrate that the PGIA model has provided fairly good guidance in identifying the Cit49 peptide as a novel T- and B-cell autoepitope in RA.

### Comparison of the present observations with the results of previous studies on citrullinated PG epitopes

Our P13 peptide pair (ATEGR/CitVRVNSAYQDK; R/Cit substitution is underlined)(S1 Table) has a 15 aa overlap with the 20 aa-long PG (aggrecan) peptide pair (VVLLVATEGR/CitVRVNSAYQDK) used in two previous studies [24][25]. Von Delwig et al [24] found significantly elevated levels of secreted and intracellular IL-17A in response to the Cit version of the 20 aa peptide as compared to the R counterpart in PBMC cultures of about 60% of the 28 RA patients tested. Law et al [25] detected significantly higher IL-6 secretion by T cells in the presence of the Cit than of the R form of the same peptide in over 60% of 21 RA patients as well as in over 60% of 6 HC subjects, whereas only a small proportion of RA patients produced greater amounts of IL-17 than HC subjects in the presence of the Cit form of this peptide [25]. However, in this particular study [25], both the RA patients and HC subjects carried HLA-DRB1 alleles encoding the so-called “shared epitope” (SE) motif that confers genetic susceptibility to RA in humans [1]. Here, we found preferential production of both IL-17 and IL-6 in response to the Cit version versus the R version of our P13 peptide (Cit:R ratios greater than 1.0) by cells from both naïve BALB/c mice (Fig 4A and 4C) and human HC subjects (Fig 7A and 7C). In our study, Cit13-stimulated PBMC also gave rise to higher proportions of Th17 and dual Th17/Th1 cells in the HC than in the RA cell cultures (Fig 8A–8C). However, with regard to IL-17 secreted by Cit13-treated cells from naïve mice or HC subjects, the concentrations of this cytokine were not elevated in comparison with the levels in Cit13-treated cultures from mice with PGIA or from humans with RA (Fig 4 legend and Fig 7B). The results obtained from our P13-treated human PBMC suggest that while the Cit form of P13 was slightly stimulatory, the R form of the same peptide was rather inhibitory for IL-17 production by HC cells, which places our observations in an intermediate position between the reports of von Delwig et al [24] and Law et al [25]. Our HC subjects were not genotyped for the presence of SE, whereas HC without the SE were not tested in the study by Law et al [25], precluding an answer to the question whether preferential recognition of the Cit13 PG epitope by T cells from HC (or RA) individuals is dependent on the SE allele.

Our P51 pair (GWLADR/CitSVRYPISKA) (S1 Table) has a 12-mer overlap with the PG peptide (AGWLADRSVRYPI) and its sequence variants used in the study by Hill et al [49]. Interestingly, when mice expressing a human HLA-DRB1*04:*01* transgene (encoding the SE in DRB1) were immunized with the wild-type (native) peptide, Hill et al [49] observed strong proliferative and IFNγ responses upon in vitro re-stimulation of draining lymph node cells with the same peptide. Immunization of the HLA-DRB1 tg mice with a variant of this peptide in which the aspartic acid residue (D) was replaced with R (AGWLARRSVRYPI) abolished these responses, but substitution of R with Cit in this peptide variant (AGWLACitRSVRYPI) led to a low degree of T-cell stimulation [49]. The latter variant (altered) pair of R and Cit peptides was used by Aggarwal et al [27] and the Cit form of the peptide was found to induce robust cell proliferation in RA PBMC cultures. It is important to note that both the R and Cit versions of this peptide represent altered human PG sequences, as the native D residue is replaced by R or Cit [27]. Here, we did not observe significant preference for the Cit form over the R form of our unaltered P51 peptide in either mice (Fig 3) or humans (S2 and S3 Figs).

Our P49 peptide pair (MDMCSAGWLADR/CitSVR) has a 9 aa overlap with our P51 pair (S1 Table), but only 8 aa homology with the altered peptide pair used by Hill et al [49] and Aggarwal et al [27]. We found that, unlike the P51 peptide pair, P49 elicited cytokine production in favor of the Cit form in spleen cell cultures of mice with PGIA (Fig 4 legend) and in PBMC cultures of both ACPA+ and ACPA- RA patients (Figs 7 and 8). In addition, circulating Abs from ACPA+ RA patients preferentially reacted with the Cit over the R version of P49 (Fig 9). In our study, high levels of anti-Cit49 Abs and high levels of Cit49-induced T-cell cytokines appeared to be associated with distinct subsets of patients within the ACPA+ RA group (Fig 10). Although expression of the SE has been genetically linked to ACPA production in RA [5][8–10], the relatively small size of our ACPA+ RA group and lack of genetic information did not allow us to determine if the production of Cit49-specific Abs was associated with a SE-expressing subset of ACPA+ RA patients. Overall, our results still suggest that P49 represents a novel PG self-epitope whose Cit form is highly immunogenic in mice with PGIA and in patients with RA.

With regard to the other PG peptides tested (P17, P33, P54, P62), we observed varying cytokine responses and Ab reactivity in both mice (Figs 3 and 5) and humans (S2–S4 Figs). Using PBMC from larger cohorts of RA patients and HC subjects would help determine if the Cit form of any of these peptides is preferentially recognized over the R form by T cells or Abs in humans.

## Conclusions

In this study, we have identified a novel citrullinated epitope in the G1 domain of human PG aggrecan. This epitope, represented by the Cit version of the peptide designated P49, is highly recognized by both T cells and Abs from mice with PGIA and from human subjects with ACPA+ RA. To our knowledge, this is the first study reporting high (although apparently independent) Ab- and T-cell reactivity with the same citrullinated self-epitope within the human PG molecule. An extended Cit version of another PG peptide, designated P13 here, was defined as a T-cell epitope in RA patients and SE-positive HC subjects in previous reports. We found preferential recognition of the Cit form over the native form of the P13 peptide by T cells from naïve BALB/c mice and from HC subjects, but not by T cells from mice with PGIA or from patients with RA. However, Abs preferentially reacting with the Cit version of P13 were rarely detected in these groups of mice or humans. We found that the magnitude of T-cell cytokine responses to the Cit form of P49 peptide was fairly high in both ACPA+ and ACPA- RA, but anti-Cit P49 Abs were present in higher quantities in the plasma of ACPA+ than ACPA- patients. However, as we demonstrate in both ACPA+ and ACPA- RA, preferential T-cell recognition of Cit P49 is not necessarily associated with the presence of circulating Abs against this peptide in the same RA patient. Since cartilage PG might be subjected to citrullination under inflammatory conditions, it may serve as a local source of Cit autoantigens (self-eptitopes) in the rheumatoid joint. Either pro-inflammatory cytokine-producing T cells or Abs reacting with Cit PG epitopes can contribute to joint destruction in RA.

## Supporting Information

S1 Fig Proliferation of spleen cells from mice with GIA in response to stimulation with peptide pools containing Cit or R peptides. Data are expressed as Cit:R ratios±SEM of SI of spleen cells from 5 mice with GIA in response to stimulation with Cit or R versions of PG and OVA peptide pools. Cit:R ratio of 1 is depicted by a dotted line. Statistical analysis was performed using Wilcoxon signed rank test (Cit:R ratios were not significantly different from 1.0). Cit:R ratios of the PG13 and other peptide pools were analyzed using Kruskal-Wallis test followed by Dunn’s multiple comparison test (#p<0.05: Any peptide pool vs PG13).
(TIF)

S2 Fig Cit:R ratios of IL-17, IFNγ and IL-6 in human PBMC supernatants induced by PG peptides P17, P33 and P51, P54 P62. Data are expressed as mean±SEM of Cit:R ratios of (A and D) IL-17 (B and E) IFNγ, and (C and F) IL-6 produced in response to stimulation with Cit or R versions of (A-C) peptides P17 and P33, or (D-F) peptides P51, P54 and P62. Cit:R ratio of 1.0 is indicated by a dotted line in each panel. Statistical analysis was performed using Wilcoxon signed rank test (*p<0.05: Cit:R ratio vs 1.0). (A-C) Ranges of sample numbers per cytokine for P17 (RA all n = 27–38; RA ACPA+ n = 21–29; RA ACPA- n = 6–11; HC n = 7–8) and for P33 (RA all n = 3–10; RA ACPA+ n = 3–7; RA ACPA- n = 0–3; HC n = 6–7). Multiple groups were compared using Kruskal Wallis test followed by Dunn’s multiple comparison test (#p<0.05: RA groups vs HC group). (D-F) Ranges of sample numbers per cytokine for P51, P54, and P62 (RA all n = 3–6 [all RA ACPA+]; RA ACPA- n = 0; HC n = 3–6). Two groups were compared using Mann-Whitney U test (no significant differences were found between the ACPA+ RA group and HC group). ND: not determined (data were available only from the ACPA+ RA group and the HC group).
(TIF)

S3 Fig Cit:R ratios of intracellular cytokines in CD4 cells in supernatants of human PBMC stimulated with peptides P17, P33, P51, P54 and P62. Data are expressed as mean±SEM of Cit:R ratios of CD4 cells containing (A and D) IL-17A, (B and E) IFNγ, or (C and F) both IL-17 and IFNγ following stimulation with (A-C) peptides P17 and P33, and (D-F) peptides P51, P54 and P62. Cit:R ratio of 1.0 is depicted by a dotted line. (A-C) Sample numbers per cytokine for P17 (RA all n = 30; RA ACPA+ n = 22; RA ACPA- n = 8; HC n = 8) and for P33 (RA all n = 6; RA ACPA+ n = 3; RA ACPA- n = 3; HC n = 7). Statistical analysis was performed using Wilcoxon signed rank test (*p<0.05: Cit:R ratio vs 1.0), and Kruskal-Wallis test followed by Dunn’s multiple comparison test (#p<0.05: RA groups vs HC group). (D-F) Ranges of sample numbers per cytokine for P51, P54, and P62 (RA all n = 6–7 [All RA ACPA+]; RA ACPA- n = 0; HC n = 4–6). Statistical analysis was done using Mann-Whitney U test (no significant differences between the ACPA+ RA group and the HC group were found).
(TIF)

S4 Fig Antibodies reacting with peptides P17, P33, P54, and P62 in plasma samples from RA patients and HC subjects. Data shown are the Cit:R ratios of ΔOD 450 nm values±SEM of IgG antibodies reacting with Cit or R versions of peptides (A) P17, (B) P33, (C) P54, and (D) P62. Cit:R ratio of 1.0 is indicated by a dotted line. Sample numbers for all peptides (RA all n = 46; RA ACPA+ n = 34; RA ACPA- n = 12; HC n = 9). Statistical analysis was performed using Wilcoxon signed rank test (Cit:R ratios were not significantly different from 1.0) and Kruskal-Wallis test followed by Dunn’s multiple comparison test (no significant differences between any of the RA groups and the HC group were found).
(TIF)

S1 Table Amino acid sequences of arginine (R)- and citrulline (Cit)-containing peptide pairs used in the study. (XLSX)

## Abbreviations

aa: amino acid
Ab: antibody
ACPA: anti-citrullinated protein Ab
CCP: citrullinated cyclic peptide
CIA: collagen-induced arthritis
CII: type II collagen
Cit: citrulline (or citrulline-containing protein/peptide)
cpm: counts per minute
ELISA: enzyme-linked immunosorbent assay
GIA: proteoglycan G1-domain-induced arthritis
HLA: human leukocyte antigen
IL-6: interleukin-6
IL-17: interleukin-17
IFNγ: interferon gamma
mAb: monoclonal Ab
MHC: major histocompatibility complex
OD: optical density
OVA: ovalbumin
P: peptide (R and Cit pair)
PAD: peptidyl arginine deiminase
PBMC: peripheral blood mononuclear cell
PBS: phosphate-buffered saline
PG: proteoglycan (aggrecan)
PGCit: citrulline-containing PG peptide pool
PGIA: PG-induced arthritis
PGR: arginine-containing PG peptide pool
RA: rheumatoid arthritis
R: arginine
rhG1: recombinant G1 domain of human PG
SE: shared epitope
SEM: standard error of the mean
SI: stimulation index
Th: T helper
