# Supplementary figures and images for: Immune Recognition of Citrullinated Proteoglycan Aggrecan Epitopes in Mice with Proteoglycan-Induced Arthritis and in Patients with Rheumatoid Arthritis
Source: PLoS One. 2016 Jul 28;11(7):e0160284. doi: 10.1371/journal.pone.0160284 (PMC4965111; doi:10.1371/journal.pone.0160284)

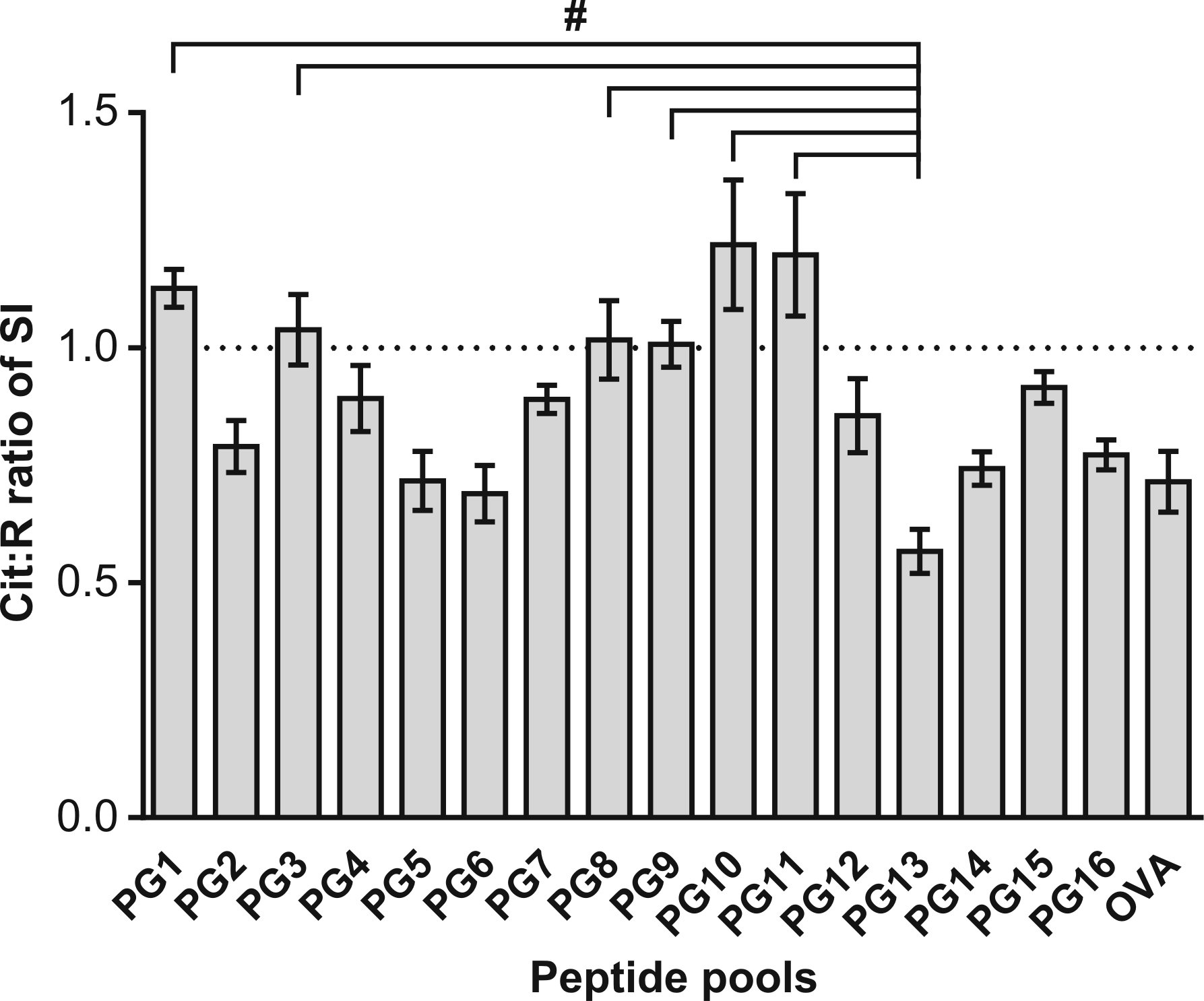

Supplement: S1 Fig — Data are expressed as Cit:R ratios±SEM of SI of spleen cells from 5 mice with GIA in response to stimulation with Cit or R versions of PG and OVA peptide pools. Cit:R ratio of 1 is depicted by a dotted line. Statistical analysis was performed using Wilcoxon signed rank test (Cit:R ratios were not significantly different from 1.0). Cit:R ratios of the PG13 and other peptide pools were analyzed using Kruskal-Wallis test followed by Dunn’s multiple comparison test (#p<0.05: Any peptide pool vs PG13). (TIF) [file pone.0160284.s001.tif]

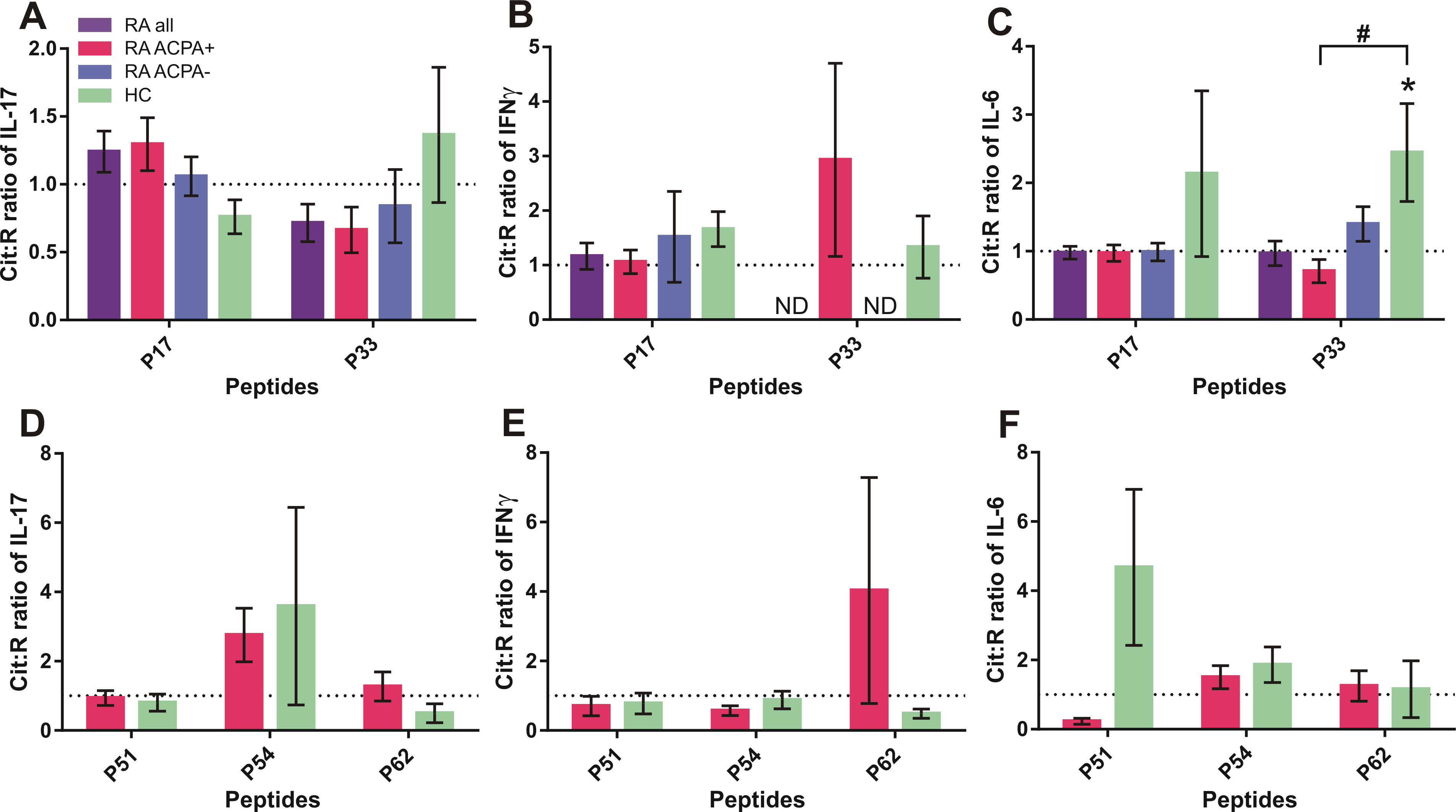

Supplement: S2 Fig — Data are expressed as mean±SEM of Cit:R ratios of (A and D) IL-17 (B and E) IFNγ, and (C and F) IL-6 produced in response to stimulation with Cit or R versions of (A-C) peptides P17 and P33, or (D-F) peptides P51, P54 and P62. Cit:R ratio of 1.0 is indicated by a dotted line in each panel. Statistical analysis was performed using Wilcoxon signed rank test (*p<0.05: Cit:R ratio vs 1.0). (A-C) Ranges of sample numbers per cytokine for P17 (RA all n = 27–38; RA ACPA+ n = 21–29; RA ACPA- n = 6–11; HC n = 7–8) and for P33 (RA all n = 3–10; RA ACPA+ n = 3–7; RA ACPA- n = 0–3; HC n = 6–7). Multiple groups were compared using Kruskal Wallis test followed by Dunn’s multiple comparison test (#p<0.05: RA groups vs HC group). (D-F) Ranges of sample numbers per cytokine for P51, P54, and P62 (RA all n = 3–6 [all RA ACPA+]; RA ACPA- n = 0; HC n = 3–6). Two groups were compared using Mann-Whitney U test (no significant differences were found between the ACPA+ RA group and HC group). ND: not determined (data were available only from the ACPA+ RA group and the HC group). (TIF) [file pone.0160284.s002.tif]

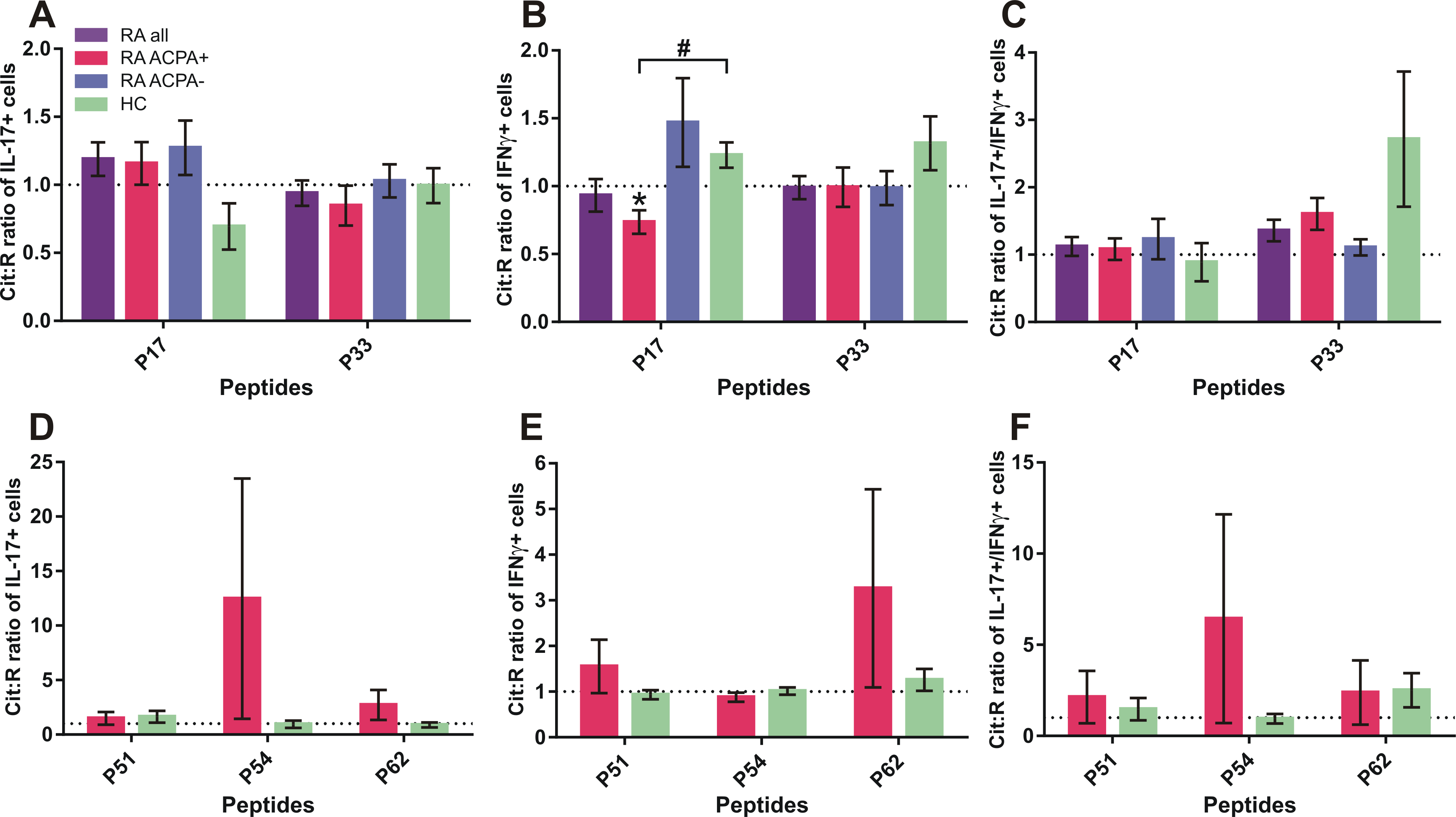

Supplement: S3 Fig — Data are expressed as mean±SEM of Cit:R ratios of CD4 cells containing (A and D) IL-17A, (B and E) IFNγ, or (C and F) both IL-17 and IFNγ following stimulation with (A-C) peptides P17 and P33, and (D-F) peptides P51, P54 and P62. Cit:R ratio of 1.0 is depicted by a dotted line. (A-C) Sample numbers per cytokine for P17 (RA all n = 30; RA ACPA+ n = 22; RA ACPA- n = 8; HC n = 8) and for P33 (RA all n = 6; RA ACPA+ n = 3; RA ACPA- n = 3; HC n = 7). Statistical analysis was performed using Wilcoxon signed rank test (*p<0.05: Cit:R ratio vs 1.0), and Kruskal-Wallis test followed by Dunn’s multiple comparison test (#p<0.05: RA groups vs HC group). (D-F) Ranges of sample numbers per cytokine for P51, P54, and P62 (RA all n = 6–7 [All RA ACPA+]; RA ACPA- n = 0; HC n = 4–6). Statistical analysis was done using Mann-Whitney U test (no significant differences between the ACPA+ RA group and the HC group were found). (TIF) [file pone.0160284.s003.tif]

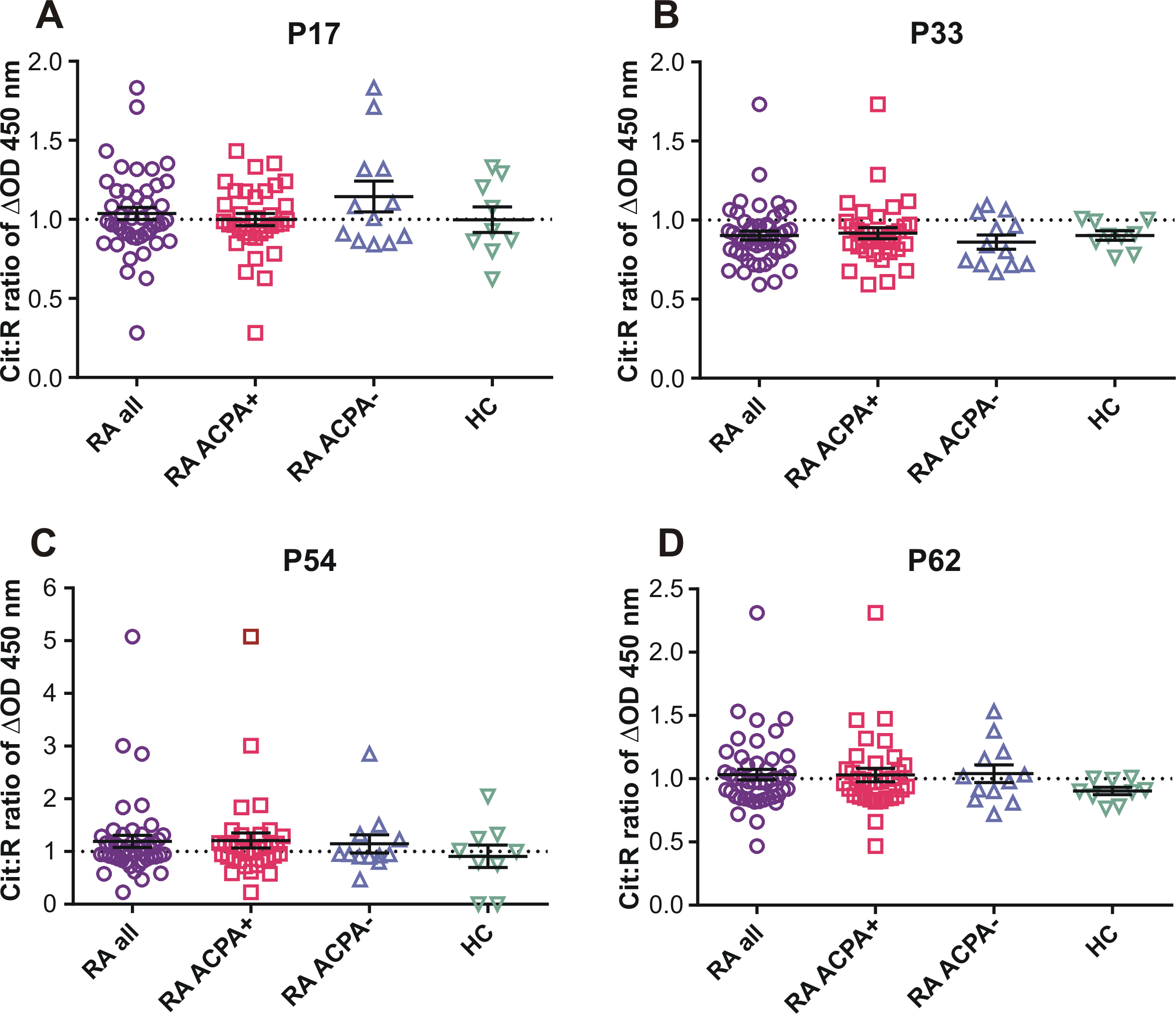

Supplement: S4 Fig — Data shown are the Cit:R ratios of ΔOD 450 nm values±SEM of IgG antibodies reacting with Cit or R versions of peptides (A) P17, (B) P33, (C) P54, and (D) P62. Cit:R ratio of 1.0 is indicated by a dotted line. Sample numbers for all peptides (RA all n = 46; RA ACPA+ n = 34; RA ACPA- n = 12; HC n = 9). Statistical analysis was performed using Wilcoxon signed rank test (Cit:R ratios were not significantly different from 1.0) and Kruskal-Wallis test followed by Dunn’s multiple comparison test (no significant differences between any of the RA groups and the HC group were found). (TIF) [file pone.0160284.s004.tif]
